# Correlation between plasma aldosterone concentration and bone mineral density in middle-aged and elderly hypertensive patients: potential impact on osteoporosis and future fracture risk

**DOI:** 10.3389/fendo.2024.1373862

**Published:** 2024-05-14

**Authors:** Shuaiwei Song, Xintian Cai, Junli Hu, Qing Zhu, Di Shen, Mulalibieke Heizhati, Wenbo Yang, Jing Hong, Nanfang Li

**Affiliations:** Hypertension Center of People’s Hospital of Xinjiang Uygur Autonomous Region, Xinjiang Hypertension Institute, NHC Key Laboratory of Hypertension Clinical Research, Key Laboratory of Xinjiang Uygur Autonomous Region, Hypertension Research Laboratory, Xinjiang Clinical Medical Research Center for Hypertension (Cardio-Cerebrovascular) Diseases, Xinjiang, Urumqi, China

**Keywords:** hypertension, plasma aldosterone concentration, bone mineral density, FRAX score, osteoporosis

## Abstract

**Background:**

Previous studies have suggested that aldosterone may play a major role in calcium-phosphorus homeostasis and bone metabolism. However, the relationship between plasma aldosterone concentrations (PAC) and bone mineral density (BMD) in middle-aged and elderly hypertensive patients remains unclear. Therefore, this study sought to investigate the relationship between PAC levels and BMD and explore PAC’s potential impact on osteoporosis and future fracture risk in hypertensive patients.

**Methods:**

Our study included a total of 1430 participants. Associations are tested using multiple linear and logistic regression models. Nonlinearity was investigated using the restricted cubic spline (RCS). We also performed mediating analyses to assess mediating factors mediating the relationship between PAC and osteoporosis.

**Results:**

The multiple linear regression showed a negative correlation between PAC and BMD and was generally positively associated with FRAX scores. Meanwhile, logistic regression analyses indicated that osteoporosis was highly correlated with PAC levels. In addition, a clear non-linear dose-response relationship was also shown in the constructed RCS model. Finally, mediation analyses showed that serum potassium played an important role in the development of osteoporosis.

**Conclusion:**

This study demonstrates that elevated PAC levels are strongly associated with decreased BMD, increased prevalence of osteoporosis, and the risk of future fractures in middle-aged and elderly hypertensive patients. Further studies are needed to confirm this relationship and reveal its underlying mechanisms.

## Highlights

- Study demonstrates for the first time the effect of plasma aldosterone concentration on bone health in middle-aged and elderly hypertensive patients.
- Plasma aldosterone concentration is significantly negatively correlated with bone mineral density in middle-aged and elderly hypertensive patients.
- Threshold effect: Plasma aldosterone concentration greater than 14 ng/dL is associated with significantly increased risk of osteoporosis and future fractures.
- Mediation analysis: serum potassium plays an important mediating role in the relationship between plasma aldosterone concentration and osteoporosis.

## 1 Introduction

Osteoporosis is a highly prevalent chronic disease described as decreased bone mineral density (BMD) (1). Both osteoporosis and hypertension are prevalent and frequently coexist as comorbid conditions in middle-aged and elderly people over the age of 40 (2). Especially in women, osteoporosis is particularly common as estrogen levels drop due to changes in menopausal status. Moreover, the prevalence of osteoporosis and hypertension (individually or in combination) is expected to significantly increase with the aging population and the extension of life expectancy (3, 4). According to a 2019 study, most people with hypertension have significantly higher rates of BMD reduction than healthy people, and in the long run, people with hypertension are more likely to develop osteoporosis (5, 6). Therefore, it is crucial to identify the causes of osteoporosis in middle-aged and elderly hypertensive patients and take early preventive measures.

Previous studies on the etiology of bone loss and osteoporosis have mostly attributed it to vitamin D deficiency, declining estrogen, glucocorticoid use, and prolonged inflammatory stimuli, while the role of aldosterone has rarely been mentioned (7–10). Aldosterone is a salt-preserving hormone secreted by the glomerular layer of the adrenal cortex (11). Several previous studies have found that excessive aldosterone production is an important risk factor for cardiovascular, renal, and metabolic diseases (12–14). However, in recent years, it has begun to be suggested that it may affect calcium metabolism and mineralocorticoid receptors have been found in human osteoblasts and osteoclasts (15, 16). Furthermore, animal experiments have also shown that disorders of bone metabolism due to excessive aldosterone secretion are associated with reduced bone mass and bone strength (17, 18). Primary aldosteronism (PA) is an adrenal gland disorder characterized by excessive secretion of aldosterone and is one of the principal types of secondary hypertension (19). Some recent evidence has found that PA is associated with an increased risk of impaired bone mass, most commonly manifested by reduced BMD and increased fracture risk (20–22). However, the relationship between aldosterone and BMD in all hypertensive populations and whether it increases the risk of osteoporosis and future fractures in patients has never been studied and is unclear. Therefore, it is worth investigating the relationship between plasma aldosterone concentration (PAC) and BMD in middle-aged and elderly hypertensive patients and exploring its potential impact on bone health, thus providing a new theoretical basis for early prevention and therapy development for osteoporosis and fractures.

Accordingly, based on the gaps in current research, this study aimed to assess the relationship between PAC and BMD in a hypertensive population as well as explore its potential impact on osteoporosis and future fracture risk. Mediation analyses were also performed to explore the mediating effects of the relevant indicators in the relationship between PAC and osteoporosis, thus providing epidemiological evidence for subsequent mechanistic studies.

## 2 Materials and methods

### 2.1 Study population

**Figure 1:**
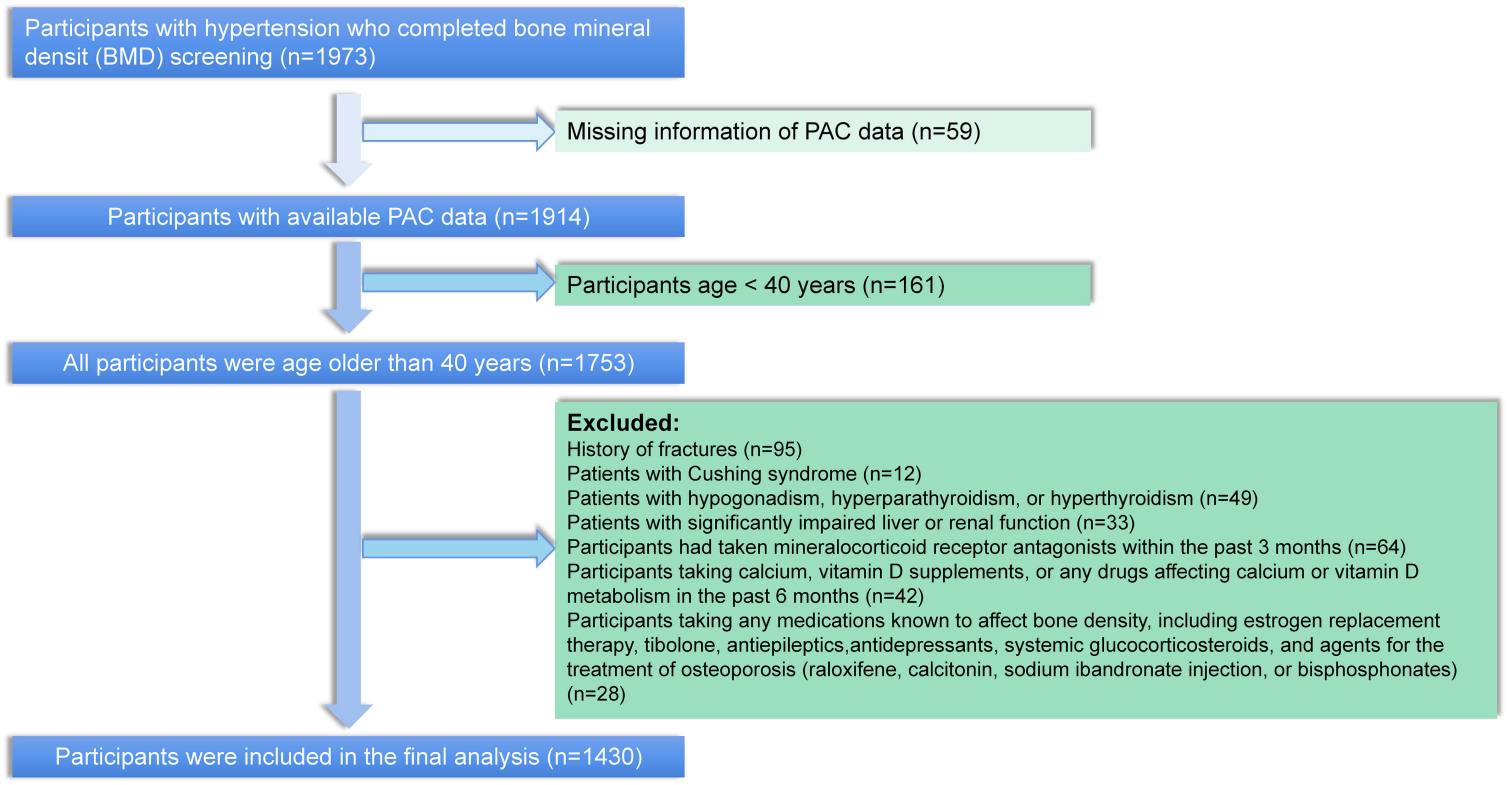
The flow chart of participant selection.

Participants in this study were selected from patients diagnosed with hypertension at the Xinjiang Hypertension Center from January 2021 to July 2023. A total of 14939 participants were diagnosed with hypertension during this period, of whom 1914 completed testing for PAC and BMD. We excluded participants younger than 40 years of age, as well as patients with a previous history of fracture, cushing’s syndrome, hypogonadism, hyperparathyroidism, hyperthyroidism, and severely impaired hepatic and renal function. In addition, to further exclude the effect of mineralocorticoid receptor antagonists on PAC measurements, we further excluded participants who had taken salocorticoid receptor antagonists in the past 3 months prior to PAC measurement. Participants taking long-term steroids, mineralocorticoid receptor antagonists, calcium supplements or vitamin D complexes, and medications that would affect BMD were also excluded. In total, 1430 participants fit the above criteria and were included for analysis (**Figure 1**).

### 2.2 Data collection and definitions

Demographic, clinical history, lifestyle, physical examination, medication history, and laboratory data were collected by electronic medical records. Detailed measurements of height, weight, smoking status, alcohol consumption, and blood pressure are provided in the **Supplementary Material**. Blood samples were collected from all participants between 8:00am and 11:00am the next day after a night of fasting, after the patient had walked for at least 2 hours and sat for 30 minutes. Laboratory parameters include alanine transaminase (ALT), aspartate transaminase (AST), creatinine (Cr), total cholesterol (TC), triglycerides (TG), high-density lipoprotein (HDL-C), low-density lipoprotein cholesterol (LDL-C), bone alkaline phosphatase (ALP), thyroid stimulating hormone (TSH), fasting plasma glucose (FPG), serum potassium, calcium, phosphorus, sodium, 24-h urinary potassium, 24-h urinary calcium, 24-h urinary phosphorus, 24-h urinary sodium, parathyroid hormone (PTH), and 25-hydroxyvitamin D were measured by fully automated biochemical analyzer. The PAC was measured by radioimmunoassay (DSL-8600; DSL, Webster, TX). Hormone measurements are based on current guidelines and previous studies conducted by our Center (23–25). Details on the specific measurements are described in the **Supplementary Materials**. Finally, the definitions of the various diseases are also detailed in the **Supplementary Materials**.

### 2.3 Evaluation of BMD

BMD was evaluated using dual-energy X-ray absorptiometry (DXA) scans. The sites of assessment included the femoral regions and the lumbar spine. Technologists who were certified radiology technologists conducted the DXA scans using the bone densitometer (Horizon Wi S/N302999M, Hologic, MA, USA). Specific details about the measurements can be found in the **Supplementary Materials**.

### 2.4 FRAX scores

FRAX scores were based on several risk factors for fracture. A China-specific FRAX assessment tool algorithm was used to determine the 10-year probability of major osteoporotic fracture (MOF) and hip fracture (HF) (26). Detailed information was available on the FRAX website (www.shef.ac.uk./FRAX).

### 2.5 Main outcome

T-scores were calculated for each site based on DXA, and osteoporosis was defined as a BMD T-score below -2.5 standard deviation of the reference value in any region of the femur and lumbar spine (27). The probability of participants’ risk of MOF and HF in the next ten years was calculated based on the China-specific FRAX score (26, 28).

### 2.6 Statistical analysis

Multiple linear regression models were used to analyze the relationship between PAC levels and BMD and the FRAX scores. The odds ratio (OR) for osteoporosis was assessed by multiple logistic regression analyses. Moreover, we used the restricted cubic spline (RCS) to assess the presence of nonlinear relationships. Two-stage analysis using RCS curve turning points to further explain non-linear relationships. Subgroup analyses were done, to assess the relationship between the different stratification factors. To test the robustness of the findings, several additional sensitivity analyses were conducted. Finally, we performed mediation analyses to test the mediating effects of potential mediators in the association of PAC with osteoporosis. Details on statistical analysis are provided in the **Supplementary Materials**.

All data were analyzed using R 4.2.2. Statistical significance was accepted for two-sided P < 0.05.

## 3 Results

### 3.1 Characteristics of participants

A total of 1430 participants were included in the study. Baseline characteristics of the study patients according to the PAC tertiles are presented in **Table 1**. Individuals with a higher PAC level were more likely to be female, current non-smokers, and non-drinkers than those with a lower PAC level. The levels of blood pressure, HDL, 24-h urinary potassium, 24-h urinary calcium, PTH, Plasma renin activity (PRA), and Aldosterone-renin ratio (ARR), as well as the rates of use of statins, calcium channel blockers, and a history of PA, increased significantly across the tertiles of PAC levels. Furthermore, the risk of MOF and HF also gradually increased in the three groups. In contrast, the BMI, serum potassium, serum phosphorus, 24-h urinary sodium, 25-hydroxy vitamin D, and DXA BMD T score all showed significant decreases across different levels of PAC.

**Table 1:** Characteristics of the study population based on PAC tertiles.

Tertiles of PAC	Tertile 1	Tertile 2	Tertile 3	P-value
	<11.92 (ng/dL)	11.92–16.52 (ng/dL)	>16.52 (ng/dL)	
Number of subjects (n)	476	476	478	
Age (years)	57.35 ± 10.70	56.01 ± 10.27	56.96 ± 12.00	0.156
Sex (%)				<0.001
Female	230 (48.32%)	233 (48.95%)	295 (61.72%)	
Male	246 (51.68%)	243 (51.05%)	183 (38.28%)	
BMI (kg/m^2^)	27.07 ± 3.93	27.00 ± 3.79	26.46 ± 3.71	0.025
SBP (mmHg)	144.79 ± 18.02	142.85 ± 15.13	147.27 ± 18.32	<0.001
DBP (mmHg)	85.36 ± 12.43	86.76 ± 11.15	88.03 ± 13.19	0.004
Current smoking (%)	113 (23.74%)	129 (27.10%)	89 (18.62%)	0.008
Current drinking (%)	86 (18.07%)	98 (20.59%)	68 (14.23%)	0.034
Medical history				
PA (%)	54 (11.34%)	109 (22.9%)	110 (23.02)	<0.001
DM (%)	191 (40.13%)	158 (33.19%)	153 (32.01%)	0.018
CHD (%)	25 (5.25%)	29 (6.09%)	38 (7.95%)	0.221
Cancer (%)	22 (4.62%)	22 (4.62%)	31 (6.49%)	0.329
Menopausal (%)	170 (35.71%)	163 (34.24%)	237 (49.58%)	<0.001
Laboratory tests				
ALT (U/L)	22.87 (15.95–34.25)	24.00 (16.00–37.00)	22.30 (16.00–34.29)	0.669
AST (U/L)	20.00 (16.00–26.00)	20.42 (16.71–26.25)	20.67 (16.33–26.00)	0.996
Cr (umol/L)	63.84 ± 16.75	64.19 ± 16.35	64.87 ± 17.72	0.637
TC (mmol/L)	4.60 ± 1.11	4.59 ± 1.08	4.64 ± 1.09	0.747
TG (mmol/L)	1.61 (1.12–2.51)	1.69 (1.18–2.57)	1.57 (1.13–2.35)	0.388
HDL-C (mg/dL)	1.09 ± 0.26	1.11 ± 0.28	1.15 ± 0.28	0.004
LDL-C (mg/dL)	2.70 (2.09–3.37)	2.71 (2.14–3.31)	2.77 (2.16–3.37)	0.567
ALP (U/L)	80.00 (62.28–104.00)	79.74 (61.21–99.88)	83.09 (64.00–104.72)	0.051
TSH (uIU/mL)	2.21 (1.42–3.42)	2.12 (1.51–3.48)	2.24 (1.44–3.42)	0.859
FPG (mmol/L)	6.13 ± 2.27	5.80 ± 1.99	5.91 ± 2.17	0.052
Serum potassium (mmol/L)	4.08 ± 0.32	3.95 ± 0.28	3.63 ± 0.29	<0.001
Serum calcium (mmol/L)	2.41 ± 0.68	2.43 ± 0.81	2.30 ± 0.55	0.006
Serum phosphorus (mmol/L)	1.20 ± 0.18	1.16 ± 0.16	1.09 ± 0.17	<0.001
Serum sodium (mmol/L)	140.73 ± 2.75	140.90 ± 2.71	140.64 ± 3.35	0.409
24-h urinary potassium (mmol/L)	30.37 (24.93–37.92)	36.42 (29.77–44.73)	43.95 (33.43–52.17)	<0.001
24-h urinary calcium (mmol/L)	3.95 (2.63–5.75)	4.53 (3.21–6.12)	5.91 (4.06–7.69)	<0.001
24-h urinary phosphorus (mmol/L)	16.83 (11.51–21.78)	17.57 (12.77–23.46)	17.20 (12.46–22.96)	0.112
24-h urinary sodium (mmol/L)	141.47 (98.15–199.71)	142.23 (96.80–191.35)	122.33 (84.74–160.72)	<0.001
PTH (pg/ml)	39.00 (27.12–53.28)	45.85 (32.77–60.20)	64.05 (44.35–80.46)	<0.001
25-hydroxyvitamin D (nmol/L)	20.73 (12.81–30.44)	19.98 (13.54–29.81)	17.06 (10.70–26.95)	<0.001
PRA (ng/mL/h)	1.07 (0.48–2.55)	1.18 (0.51–2.79)	2.00 (0.63–3.97)	<0.001
ARR	8.42 (3.78–19.51)	10.86 (4.71–25.13)	10.99 (5.61–30.86)	<0.001
Medications				
Statins (%)	65 (13.66%)	69 (14.50%)	92 (19.25%)	0.038
Aspirins (%)	60 (12.61%)	58 (12.18%)	76 (15.90%)	0.186
Diuretics (%)	36 (7.56%)	49 (10.29%)	53 (11.09%)	0.154
Beta-blockers (%)	71 (14.92%)	67 (14.08%)	81 (16.95%)	0.449
Calcium channel blockers (%)	212 (44.54%)	240 (50.42%)	261 (54.60%)	0.008
ACEIs/ARBs (%)	159 (33.40%)	176 (36.97%)	193 (40.38%)	0.083
Oral hypoglycemic agents (%)	144 (30.25%)	115 (24.16%)	116 (24.27%)	0.050
Insulin (%)	35 (7.35%)	34 (7.14%)	32 (6.69%)	0.921
DXA BMD T-scores				
Lumbar 1	-0.26 ± 1.64	-0.62 ± 1.51	-1.63 ± 1.43	<0.001
Lumbar 2	-0.20 ± 1.65	-0.63 ± 1.51	-1.57 ± 1.47	<0.001
Lumbar 3	-0.12 ± 1.72	-0.58 ± 1.53	-1.61 ± 1.56	<0.001
Lumbar 4	0.03 ± 1.75	-0.45 ± 1.58	-1.53 ± 1.51	<0.001
Neck	-0.30 ± 1.15	-0.69 ± 0.93	-1.39 ± 0.93	<0.001
Wards	-0.52 ± 1.29	-0.94 ± 1.05	-1.74 ± 1.08	<0.001
Total	0.48 ± 1.04	0.01 ± 0.87	-0.81 ± 0.96	<0.001
FRAX scores (%)				
MOF	2.78 ± 1.92	3.15 ± 2.05	5.44 ± 3.83	<0.001
HF	0.80 ± 1.33	1.03 ± 1.46	2.71 ± 3.28	<0.001
**Osteoporosis (%)**	68 (14.29%)	85 (17.86%)	208 (43.51%)	<0.001

Data are presented as mean ± standard deviation, median (interquartile range), or as numbers, and percentages.

BMI, body mass index; SBP, systolic blood pressure; DBP, diastolic blood pressure; PA, primary aldosteronism; DM, diabetes mellitus; CHD, coronary heart disease; ALT, alanine transaminase; AST, aspartate transaminase; Cr, creatinine; TC, total cholesterol; TG, triglyceride; HDL-C, high-density lipoprotein cholesterol; LDL-C, low-density lipoprotein cholesterol; ALP, alkaline phosphatase; TSH, thyroid stimulating hormone; FPG, fasting plasma glucose; PTH, parathyroid hormone; PRA, Plasma renin activity; ARR, Aldosterone-renin ratio; PAC, plasma aldosterone concentration; ARBs, angiotensin receptor blockers; ACEIs, angiotensin-converting enzyme inhibitors; BMD, bone mineral density; Neck, neck of the femur; Wards, Ward’s triangle; Total, total femur; MOF, major osteoporotic fracture; HF, hip fracture.

### 3.2 Relationship between PAC levels and BMD

In the multiple linear regression model, PAC is significantly negatively correlated with BMD (**Table 2**). Furthermore, when PAC is converted into a categorical variable, the negative correlation between PAC and BMD still exists. Compared to participants in group T1, participants in groups T2 and T3 exhibited significantly lower BMD T-scores in lumbar 1–4, total femur (Toal), Ward’s triangle (Wards), and femoral neck (Neck). After adjusting for all covariates in Model 5, the results indicated that the BMD T-scores at all sites in group T3 remained significantly lower than those in group T1.

**Table 2:** Relationship between PAC levels and BMD.

Exposure	Model 1	Model 2	Model 3	Model 4	Model 5
	β (95% CI) P	β (95% CI) P	β (95% CI) P	β (95% CI) P	β (95% CI) P
Lumbar 1					
PAC (per 1-ng/dL increase)	-0.07 (-0.08, -0.06) <0.001	-0.07 (-0.08, -0.06) <0.001	-0.05 (-0.07, -0.04) <0.001	-0.05 (-0.07, -0.04) <0.001	-0.06 (-0.07, -0.04) <0.001
Tertiles of PAC					
Tertile 1	Reference	Reference	Reference	Reference	Reference
Tertile 2	-0.36 (-0.55, -0.16) <0.001	-0.37 (-0.56, -0.19) <0.001	-0.36 (-0.55, -0.17) <0.001	-0.38 (-0.57, -0.19) <0.001	-0.38 (-0.57, -0.19) <0.001
Tertile 3	-1.38 (-1.57, -1.18) <0.001	-1.27 (-1.46, -1.08) <0.001	-1.12 (-1.35, -0.90) <0.001	-1.13 (-1.36, -0.90) <0.001	-1.13 (-1.36, -0.90) <0.001
P for trend	<0.001	<0.001	<0.001	<0.001	<0.001
Lumbar 2					
PAC (per 1-ng/dL increase)	-0.07 (-0.08, -0.06) <0.001	-0.06 (-0.08, -0.05) <0.001	-0.05 (-0.06, -0.03) <0.001	-0.05 (-0.07, -0.04) <0.001	-0.05 (-0.07, -0.04) <0.001
Tertiles of PAC					
Tertile 1	Reference	Reference	Reference	Reference	Reference
Tertile 2	-0.44 (-0.63, -0.24) <0.001	-0.46 (-0.65, -0.28) <0.001	-0.45 (-0.63, -0.26) <0.001	-0.47 (-0.66, -0.28) <0.001	-0.47 (-0.66, -0.28) <0.001
Tertile 3	-1.38 (-1.57, -1.18) <0.001	-1.26 (-1.45, -1.08) <0.001	-1.11 (-1.33, -0.88) <0.001	-1.12 (-1.35, -0.90) <0.001	-1.13 (-1.36, -0.90) <0.001
P for trend	<0.001	<0.001	<0.001	<0.001	<0.001
Lumbar 3					
PAC (per 1-ng/dL increase)	-0.07 (-0.09, -0.06) <0.001	-0.07 (-0.08, -0.06) <0.001	-0.05 (-0.06, -0.03) <0.001	-0.05 (-0.07, -0.04) <0.001	-0.05 (-0.07, -0.04) <0.001
Tertiles of PAC					
Tertile 1	Reference	Reference	Reference	Reference	Reference
Tertile 2	-0.46 (-0.66, -0.25) <0.001	-0.48 (-0.67, -0.28) <0.001	-0.44 (-0.64, -0.25) <0.001	-0.47 (-0.67, -0.27) <0.001	-0.48 (-0.68, -0.29) <0.001
Tertile 3	-1.49 (-1.69, -1.29) <0.001	-1.36 (-1.55, -1.16) <0.001	-1.14 (-1.38, -0.91) <0.001	-1.16 (-1.40, -0.93) <0.001	-1.18 (-1.42, -0.95) <0.001
P for trend	<0.001	<0.001	<0.001	<0.001	<0.001
Lumbar 4					
PAC (per 1-ng/dL increase)	-0.08 (-0.10, -0.07) <0.001	-0.08 (-0.09, -0.07) <0.001	-0.06 (-0.07, -0.04) <0.001	-0.06 (-0.08, -0.05) <0.001	-0.06 (-0.08, -0.05) <0.001
Tertiles of PAC					
Tertile 1	Reference	Reference	Reference	Reference	Reference
Tertile 2	-0.48 (-0.68, -0.27) <0.001	-0.49 (-0.69, -0.29) <0.001	-0.45 (-0.65, -0.25) <0.001	-0.48 (-0.68, -0.28) <0.001	-0.48 (-0.68, -0.28) <0.001
Tertile 3	-1.56 (-1.76, -1.35) <0.001	-1.43 (-1.63, -1.23) <0.001	-1.19 (-1.43, -0.95) <0.001	-1.20 (-1.45, -0.96) <0.001	-1.21 (-1.45, -0.97) <0.001
P for trend	<0.001	<0.001	<0.001	<0.001	<0.001
Neck					
PAC (per 1-ng/dL increase)	-0.06 (-0.07, -0.05) <0.001	-0.06 (-0.07, -0.05) <0.001	-0.04 (-0.05, -0.03) <0.001	-0.04 (-0.05, -0.03) <0.001	-0.04 (-0.05, -0.03) <0.001
Tertiles of PAC					
Tertile 1	Reference	Reference	Reference	Reference	Reference
Tertile 2	-0.40 (-0.52, -0.27) <0.001	-0.44 (-0.56, -0.32)	-0.41 (-0.53, -0.29)	-0.42 (-0.54, -0.30)	-0.42 (-0.54, -0.30)
Tertile 3	-1.09 (-1.22, -0.96) <0.001	-1.08 (-1.20, -0.95) <0.001	-0.90 (-1.04, -0.76) <0.001	-0.89 (-1.03, -0.74) <0.001	-0.88 (-1.02, -0.73) <0.001
P for trend	<0.001	<0.001	<0.001	<0.001	<0.001
Wards					
PAC (per 1-ng/dL increase)	-0.07 (-0.08, -0.06) <0.001	-0.07 (-0.08, -0.06) <0.001	-0.05 (-0.06, -0.04) <0.001	-0.05 (-0.06, -0.04) <0.001	-0.05 (-0.06, -0.04) <0.001
Tertiles of PAC					
Tertile 1	Reference	Reference	Reference	Reference	Reference
Tertile 2	-0.42 (-0.56, -0.27) <0.001	-0.48 (-0.62, -0.34) <0.001	-0.43 (-0.57, -0.29) <0.001	-0.46 (-0.60, -0.32) <0.001	-0.46 (-0.60, -0.32) <0.001
Tertile 3	-1.23 (-1.37, -1.08) <0.001	-1.23 (-1.37, -1.09) <0.001	-1.02 (-1.19, -0.86) <0.001	-1.01 (-1.17, -0.84) <0.001	-1.00 (-1.17, -0.84) <0.001
P for trend	<0.001	<0.001	<0.001	<0.001	<0.001
Total					
PAC (per 1-ng/dL increase)	-0.07 (-0.08, -0.06) <0.001	-0.07 (-0.08, -0.06) <0.001	-0.05 (-0.06, -0.04) <0.001	-0.05 (-0.06, -0.04) <0.001	-0.05 (-0.06, -0.04) <0.001
Tertiles of PAC					
Tertile 1	Reference	Reference	Reference	Reference	Reference
Tertile 2	-0.47 (-0.59, -0.35) <0.001	-0.51 (-0.62, -0.40) <0.001	-0.46 (-0.57, -0.35) <0.001	-0.47 (-0.58, -0.36) <0.001	-0.48 (-0.59, -0.36) <0.001
Tertile 3	-1.29 (-1.41, -1.17) <0.001	-1.25 (-1.37, -1.14) <0.001	-1.04 (-1.18, -0.91) <0.001	-1.03 (-1.16, -0.89) <0.001	-1.04 (-1.17, -0.90) <0.001
P for trend	<0.001	<0.001	<0.001	<0.001	<0.001

Model 1: no covariates were adjusted.

Model 2: age, sex, BMI, smoking status, and drinking status were adjusted.

Model 3: Model 2 plus adjustment for PA, DM, CHD, and cancer.

Model 4: Model 3 plus adjustment for ALT, AST, Cr, TC, TG, HDL-C, LDL-C, ALP, TSH, FPG, serum potassium, serum calcium, serum phosphorus, serum sodium, 24-h urinary potassium, 24-h urinary calcium,24-h urinary phosphorus, 24-h urinary sodium, PTH, and 25-hydroxyvitamin D.

Model 5: Model 4 plus adjustment for use of statins, aspirin, diuretics, beta-blockers, calcium channel blockers, ACEIs/ARBs, oral hypoglycemic agents, and insulin.

PAC, plasma aldosterone concentration; BMD, bone mineral density; Neck, neck of the femur; Wards, Ward’s triangle; Total, total femur; β, regression coefficient; CI, confidence interval.

Other abbreviations, see **Table 1**.

### 3.3 Relationship between PAC levels and FRAX score

Meanwhile, in the relationship between PAC and FRAX score, we also found a certain positive correlation between PAC and MOF, and HF (**Table 3**). As PAC levels increase, the risk of future fractures may further increase. In any model, whether MOF or HF, the risk of future fractures was significantly increased in the T2 and T3 groups compared to the reference (T1) group.

**Table 3:** Relationship between PAC levels and FRAX scores.

Exposure	Model 1	Model 2	Model 3	Model 4	Model 5
	β (95% CI) P	β (95% CI) P	β (95% CI) P	β (95% CI) P	β (95% CI) P
MOF					
PAC (per 1-ng/dL increase)	0.16 (0.14, 0.19) <0.001	0.16 (0.14, 0.18) <0.001	0.12 (0.10, 0.15) <0.001	0.13 (0.10, 0.15) <0.001	0.13 (0.10, 0.15) <0.001
Tertiles of PAC					
Tertile 1	Reference	Reference	Reference	Reference	Reference
Tertile 2	0.37 (0.02, 0.72) 0.037	0.45 (0.12, 0.77) 0.006	0.30 (-0.02, 0.63) 0.070	0.38 (0.05, 0.70) 0.022	0.38 (0.05, 0.70) 0.023
Tertile 3	2.66 (2.31, 3.01) <0.001	2.55 (2.22, 2.87) <0.001	1.97 (1.59, 2.36) <0.001	2.00 (1.61, 2.39) <0.001	2.00 (1.60, 2.39) <0.001
P for trend	<0.001	<0.001	<0.001	<0.001	<0.001
HF					
PAC (per 1-ng/dL increase)	0.13 (0.11, 0.14) <0.001	0.13 (0.11, 0.14) <0.001	0.10 (0.08, 0.12) <0.001	0.10 (0.08, 0.12) <0.001	0.10 (0.08, 0.12) <0.001
Tertiles of PAC					
Tertile 1	Reference	Reference	Reference	Reference	Reference
Tertile 2	0.23 (-0.05, 0.51) 0.105	0.26 (-0.01, 0.53) 0.062	0.15 (-0.13, 0.42) 0.287	0.21 (-0.06, 0.48) 0.127	0.21 (-0.06, 0.49) 0.125
Tertile 3	1.92 (1.64, 2.20) <0.001	1.92 (1.65, 2.20) <0.001	1.49 (1.17, 1.82) <0.001	1.52 (1.19, 1.85) <0.001	1.52 (1.19, 1.85) <0.001
P for trend	<0.001	<0.001	<0.001	<0.001	<0.001

Model 1: no covariates were adjusted.

Model 2: age, sex, BMI, smoking status, and drinking status were adjusted.

Model 3: Model 2 plus adjustment for PA, DM, CHD, and cancer.

Model 4: Model 3 plus adjustment for ALT, AST, Cr, TC, TG, HDL-C, LDL-C, ALP, TSH, FPG, serum potassium, serum calcium, serum phosphorus, serum sodium, 24-h urinary potassium, 24-h urinary calcium,24-h urinary phosphorus, 24-h urinary sodium, PTH, and 25-hydroxyvitamin D.

Model 5: Model 4 plus adjustment for use of statins, aspirin, diuretics, beta-blockers, calcium channel blockers, ACEIs/ARBs, oral hypoglycemic agents, and insulin.

PAC, plasma aldosterone concentration; MOF, major osteoporotic fracture; HF, hip fracture; β, regression coefficient; CI, confidence interval.

Other abbreviations, see **Table 1**.

### 3.4 Relationship between PAC levels and osteoporosis

Logistic regression analysis showed that osteoporosis was highly correlated with the levels of PAC (OR, 1.09; 95% confidence interval [CI], 1.07–1.12). This correlation remains significant in Model 5 adjusted for all covariates (OR, 1.07; 95% CI, 1.04–1.09). Compared with the T1 group, the ORs of osteoporosis were 1.36 (95% CI, 0.94–1.97) in the T2 group and 3.42 (95% CI, 2.29–5.09) in the T3 group (**Table 4**).

**Table 4:** Associations between PAC levels and osteoporosis.

Exposure	Model 1	Model 2	Model 3	Model 4	Model 5
	OR (95%CI) P	OR (95%CI) P	OR (95%CI) P	OR (95%CI) P	OR (95%CI) P
PAC (per 1-ng/dL increase)	1.09 (1.07, 1.12) <0.001	1.09 (1.07, 1.12) <0.001	1.06 (1.04, 1.09) <0.001	1.07 (1.04, 1.09) <0.001	1.07 (1.04, 1.09) <0.001
Tertiles of PAC					
Tertile 1	Reference	Reference	Reference	Reference	Reference
Tertile 2	1.30 (0.92, 1.85) 0.134	1.36 (0.95, 1.95) 0.087	1.28 (0.89, 1.85) 0.177	1.34 (0.93, 1.94) 0.118	1.36 (0.94, 1.97) 0.107
Tertile 3	4.62 (3.38, 6.33) <0.001	4.54 (3.27, 6.29) <0.001	3.32 (2.26, 4.88) <0.001	3.41 (2.30, 5.06) <0.001	3.42 (2.29, 5.09) <0.001
P for trend	<0.001	<0.001	<0.001	<0.001	<0.001

Model 1: no covariates were adjusted.

Model 2: age, sex, BMI, smoking status, and drinking status were adjusted.

Model 3: Model 2 plus adjustment for PA, DM, CHD, and cancer.

Model 4: Model 3 plus adjustment for ALT, AST, Cr, TC, TG, HDL-C, LDL-C, ALP, TSH, FPG, serum potassium, serum calcium, serum phosphorus, serum sodium, 24-h urinary potassium, 24-h urinary calcium,24-h urinary phosphorus, 24-h urinary sodium, PTH, and 25-hydroxyvitamin D.

Model 5: Model 4 plus adjustment for use of statins, aspirin, diuretics, beta-blockers, calcium channel blockers, ACEIs/ARBs, oral hypoglycemic agents, and insulin.

PAC, plasma aldosterone concentration; OR, odds ratio; CI, confidence interval.

Other abbreviations, see **Table 1**.

### 3.5 Nonlinear relationship and two-stage analysis

**Figure 2:**
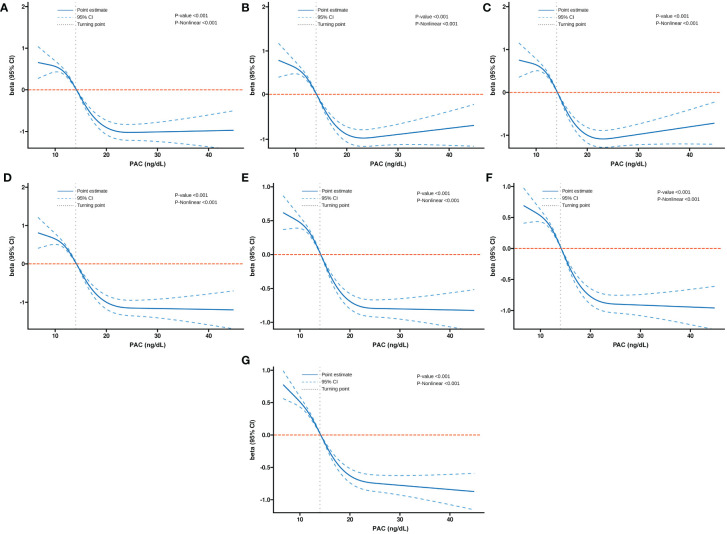
Non-linear dose-response relationship between PAC and BMD. **(A)** Lumbar 1, **(B)** Lumbar 2, **(C)** Lumbar 3, **(D)** Lumbar 4, **(E)** Neck, **(F)** Wards, and **(G)** Total.

**Figure 3:**
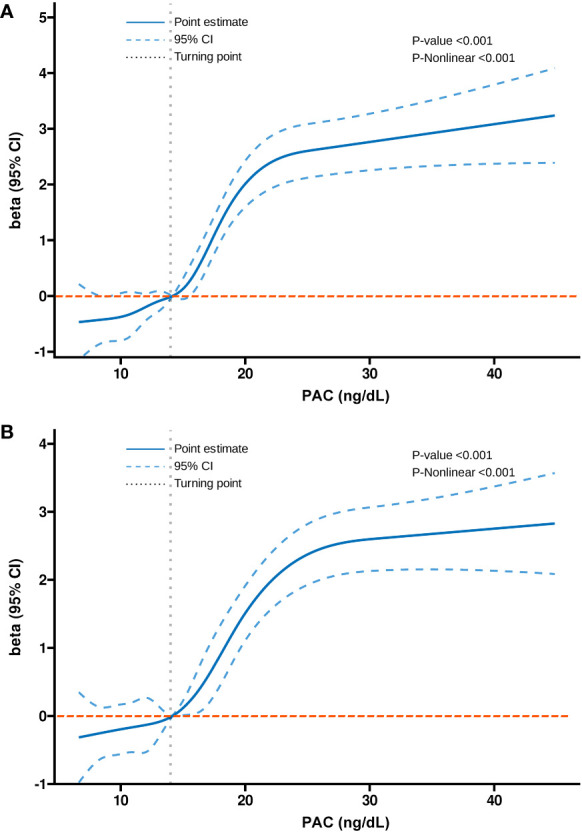
Dose-response relationship between PAC and FRAX score. **(A)** MOF and **(B)** HF.

**Figure 4:**
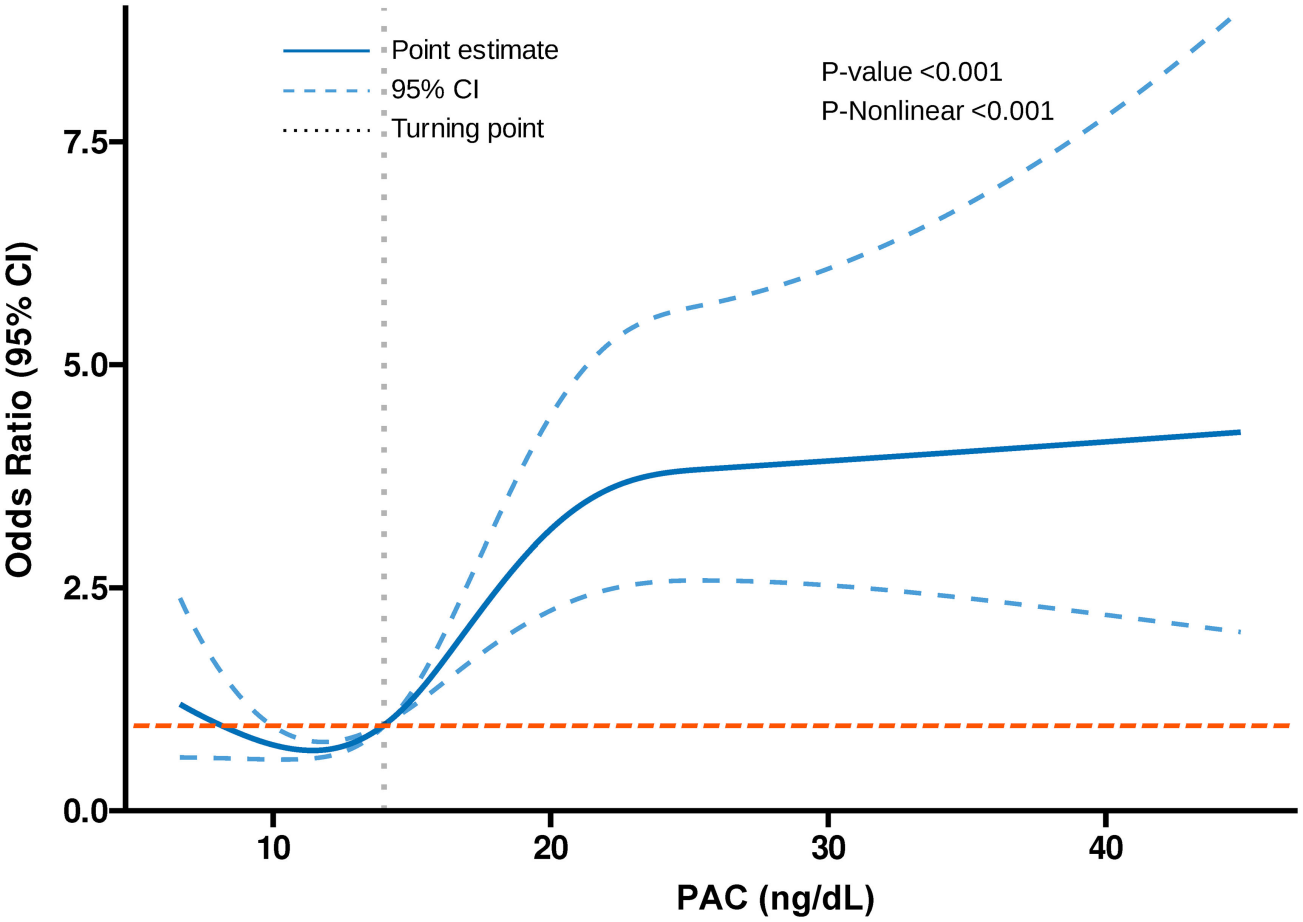
Dose-response association between PAC and risk of osteoporosis.

Additionally, we also used RCS to assess the nonlinear dose-response relationships between PAC and BMD, FRAX score, and osteoporosis. **Figures 2**–**4** show the non-linear trends of these associations from the RCS analyses. By observing the curves, we calculated the turning points and used a two-stage analysis to further explain the correlation. The results indicate that the risk of future fractures gradually increases when PAC exceeds 14 ng/dL (**Table 5**). Furthermore, in the threshold analysis for PAC and osteoporosis, it was also found that when PAC exceeds 14 ng/dL, for every unit increase in PAC, the risk of osteoporosis increases by 5%, while there is no statistically significant association when PAC is below 14 ng/dL (**Table 6**). The log-likelihood ratio test for PAC at each turning point was statistically significant (p < 0.001), suggesting that the two-stage analysis before and after the turning point is suitable for describing the relationship between PAC and MOF, HF, and osteoporosis (**Tables 5**, **6**).

**Table 5:** Analyzing the relationship between PAC levels and ten-year fracture risk using the RCS turning point.

MOF	β (95% CI) P
Turning point (ng/dL)	14
PAC < 14 ng/dL	0 (-0.07, 0.08) 0.960
PAC>14 ng/dL	0.25 (0.09, 0.16) <0.001
P for log-likelihood ratio test	<0.001
**HF**	β (95% CI)
Turning point (ng/dL)	14
PAC < 14 ng/dL	0 (-0.06, 0.05) 0.881
PAC>14 ng/dL	0.26 (0.19, 0.33) <0.001
P for log-likelihood ratio test	<0.001

Age, sex, BMI, smoking status, drinking status, PA, DM, CHD, cancer, ALT, AST, Cr, TC, TG, HDL-C, LDL-C, ALP, TSH, FPG, serum potassium, serum calcium, serum phosphorus, serum sodium, 24-h urinary potassium, 24-h urinary calcium,24-h urinary phosphorus, 24-h urinary sodium, PTH, 25-hydroxyvitamin D, statins, aspirin, diuretics, beta-blockers, calcium channel blockers, ACEIs/ARBs, oral hypoglycemic agents, and insulin were adjusted.

PAC, plasma aldosterone concentration; MOF, major osteoporotic fracture; HF, hip fracture; RCS, restricted cubic splines; β, regression coefficient; CI, confidence interval.

Other abbreviations, see **Table 1**.

**Table 6:** Analyzing the relationship between PAC and osteoporosis using the RCS turning points.

Osteoporosis	OR (95% CI) P
Turning point (ng/dL)	14
PAC < 14 ng/dL	0.90 (0.80, 1.01) 0.079
PAC>14 ng/dL	1.05 (1.03, 1.08) <0.001
P for log-likelihood ratio test	<0.001

Age, sex, BMI, smoking status, drinking status, PA, DM, CHD, cancer, ALT, AST, Cr, TC, TG, HDL-C, LDL-C, ALP, TSH, FPG, serum potassium, serum calcium, serum phosphorus, serum sodium, 24-h urinary potassium, 24-h urinary calcium,24-h urinary phosphorus, 24-h urinary sodium, PTH, 25-hydroxyvitamin D, statins, aspirin, diuretics, beta-blockers, calcium channel blockers, ACEIs/ARBs, oral hypoglycemic agents, and insulin were adjusted.

PAC, plasma aldosterone concentration; RCS, restricted cubic splines; OR, odds ratio; CI, confidence interval.

Other abbreviations, see **Table 1**.

### 3.6 Subgroup and sensitivity analysis

In subgroup analyses, the results remained consistent after stratification by sex, age, BMI, PRA, ARR, smoking status, alcohol status, and diabetes mellitus (**Tables 7**, **8**). At the same time, among women, we further stratified the analyses according to menopausal status, and the results obtained were largely in line with the general trends (**Supplementary Table S4**). In addition, because of the skewness of the PAC distribution, a natural logarithm transformation was applied to the data, and the results obtained were very stable (**Supplementary Tables S5-S7**). In the sensitivity analyses, we excluded data with missing values from the analysis and obtained essentially the same results (**Supplementary Table S8**). We also excluded participants with outliers and obtained very reliable results (**Supplementary Table S9**). Also, we excluded patients with cancer, and the results remained consistent (**Supplementary Table S10**). Additionally, participants with 25-hydroxyvitamin D < 20 nmol/L were excluded, and the results still showed a strong correlation (**Supplementary Table S11**). Furthermore, we further excluded patients with BMI > 30 kg/m^2^, and the results remained consistent (**Supplementary Table S12**). We also excluded patients with age > 75 years, and the results held up well (**Supplementary Table S13**). Finally, we again excluded patients with a previous definitive diagnosis of PA, a result that remains reliable (**Supplementary Table S14**).

**Table 7:** Effect of each stratification factor on the relationship between PAC and BMD.

BMD T-scores	Lumbar 1 β (95%CI) P	Lumbar 2 β (95%CI) P	Lumbar 3 β (95%CI) P	Lumbar 4 β (95%CI) P	Neck β (95%CI) P	Wards β (95%CI) P	Total β (95%CI) P
Sex							
Female	-0.05 (-0.05, -0.07) <0.001	-0.05 (-0.07, -0.03) <0.001	-0.05 (-0.07, -0.03) <0.001	-0.05 (-0.07, -0.03) <0.001	-0.04 (-0.06, -0.03) <0.001	-0.05 (-0.07, -0.04) <0.001	-0.05 (-0.05, -0.07) <0.001
Male	-0.07 (-0.07, -0.09) <0.001	-0.05 (-0.07, -0.03) 0.001	-0.05 (-0.07, -0.03) 0.003	-0.08 (-0.10, -0.06) <0.001	-0.04 (-0.05, -0.03) <0.001	-0.04 (-0.06, -0.03) <0.001	-0.06 (-0.07, -0.05) <0.001
Age (years)							
<60	-0.05 (-0.07, -0.03) <0.001	-0.05 (-0.07, -0.03) <0.001	-0.06 (-0.08, -0.04) <0.001	-0.06 (-0.07, -0.04) <0.001	-0.04 (-0.05, -0.03) <0.001	-0.05 (-0.06, -0.04) <0.001	-0.05 (-0.06, -0.04) <0.001
>=60	-0.07 (-0.09, -0.04) <0.001	-0.06 (-0.08, -0.03) <0.001	-0.05 (-0.08, -0.03) <0.001	-0.08 (-0.11, -0.06) <0.001	-0.05 (-0.07, -0.03) <0.001	-0.05 (-0.08, -0.03) <0.001	-0.06 (-0.07, -0.04) <0.001
BMI (kg/m^2^)							
<24	-0.08 (-0.08, -0.11) <0.001	-0.08 (-0.11, -0.04) <0.001	-0.08 (-0.12, -0.04) <0.001	-0.10 (-0.13, -0.06) <0.001	-0.05 (-0.08, -0.03) <0.001	-0.07 (-0.1, -0.04) <0.001	-0.06 (-0.08, -0.04) <0.001
>=24	-0.05 (-0.05, -0.07) <0.001	-0.05 (-0.06, -0.03) <0.001	-0.05 (-0.06, -0.03) <0.001	-0.06 (-0.08, -0.04) <0.001	-0.05 (-0.07, -0.03) <0.001	-0.04 (-0.05, -0.03) <0.001	-0.05 (-0.06, -0.04) <0.001
PRA (ng/mL/h)							
PRA<=0.5	-0.04 (-0.06, -002) <0.001	-0.05 (-0.06, -003) <0.001	-0.05 (-0.07, -003) <0.001	-0.05 (-0.07, -003) <0.001	-0.04 (-0.05, -003) <0.001	-0.05 (-0.06, -003) <0.001	-0.04 (-0.06, -004) <0.001
PRA>0.5	-0.05 (-0.06, -0.04) <0.001	-0.04 (-0.06, -003) <0.001	-0.05 (-0.06, -004) <0.001	-0.06 (-0.07, -005) <0.001	-0.04 (-0.05, -003) <0.001	-0.04 (-0.05, -004) <0.001	-0.05 (-0.06, -004) <0.001
ARR							
ARR<20	-0.04 (-0.06, -003) <0.001	-0.05 (-0.06, -003) <0.001	-0.05 (-0.07, -003) <0.001	-0.05 (-0.07, -003) <0.001	-0.04 (-0.05, -003) <0.001	-0.05 (-0.06, -004) <0.001	-0.05 (-0.06, -003) <0.001
ARR>=20	-0.05 (-0.06, -004) <0.001	-0.04 (-0.06, -003) <0.001	-0.05 (-0.06, -004) <0.001	-0.06 (-0.07, -005) <0.001	-0.04 (-0.05, -003) <0.001	-0.04 (-0.05, -003) <0.001	-0.05 (-0.06, -004) <0.001
Current smoking							
No	-0.05 (-0.07, -0.04) <0.001	-0.06 (-0.07, -0.04) <0.001	-0.06 (-0.08, -0.04) <0.001	-0.06 (-0.08, -0.04) <0.001	-0.04 (-0.05, -0.03) <0.001	-0.05 (-0.07, -0.04) <0.001	-0.06 (-0.07, -0.05) <0.001
Yes	-0.06 (-0.09, -0.04) <0.001	-0.05 (-0.07, -0.02) 0.001	-0.04 (-0.07, -0.01) 0.003	-0.08 (-0.11, -0.05) <0.001	-0.04 (-0.06, -0.02) <0.001	-0.04 (-0.06, -0.02) <0.001	-0.04 (-0.06, -0.02) <0.001
Current drinking							
No	-0.05 (-0.07, -0.04) <0.001	-0.05(-0.06, -0.03) <0.001	-0.05 (-0.06, -0.03) <0.001	-0.06 (-0.08, -0.04) <0.001	-0.04 (-0.05, -0.03) <0.001	-0.05 (-0.06, -0.04) <0.001	-0.05 (-0.06, -0.04) <0.001
Yes	-0.06 (-0.09, -0.03) <0.001	-0.06 (-0.09, -0.03) <0.001	-0.06 (-0.10, -0.03) <0.001	-0.06 (-0.09, -0.03) <0.001	-0.03 (-0.05, -0.01) 0.002	-0.03 (-0.06, -0.01) <0.001	-0.04 (-0.06, -0.02) <0.001
DM							
No	-0.05 (-0.07, -0.04) <0.001	-0.05 (-0.06, -0.03) <0.001	-0.05 (-0.06, -0.03) <0.001	-0.06 (-0.08, -0.04) <0.001	-0.04 (-0.06, -0.03) <0.001	-0.05 (-0.06, -0.03) <0.001	-0.05 (-0.06, -0.04) <0.001
Yes	-0.06 (-0.09, -0.03) <0.001	-0.06 (-0.09, -0.03) <0.001	-0.08 (-0.11, -0.05) <0.001	-0.07 (-0.1, -0.04) <0.001	-0.03 (-0.05, -0.01) <0.001	-0.08 (-0.07, -0.03) <0.001	-0.05 (-0.07, -0.04) <0.001

Age, sex, BMI, smoking status, drinking status, PA, DM, CHD, cancer, ALT, AST, Cr, TC, TG, HDL-C, LDL-C, ALP, TSH, FPG, serum potassium, serum calcium, serum phosphorus, serum sodium, 24-h urinary potassium, 24-h urinary calcium,24-h urinary phosphorus, 24-h urinary sodium, PTH, 25-hydroxyvitamin D, statins, aspirin, diuretics, beta-blockers, calcium channel blockers, ACEIs/ARBs, oral hypoglycemic agents, and insulin were adjusted.

PAC, plasma aldosterone concentration; BMD, bone mineral density; PRA, Plasma renin activity; ARR, Aldosterone-renin ratio; Neck, neck of the femur; Wards, Ward’s triangle; Total, total femur; MOF, major osteoporotic fracture; HF, hip fracture; β, regression coefficient; CI, confidence interval.

Other abbreviations, see **Table 1**.

**Table 8:** Effect of each stratification factor on the relationship between PAC and osteoporosis.

Osteoporosis	OR 95%CI	P value
Sex		
Female	1.05 (1.02, 1.09)	0.001
Male	1.09 (1.04, 1.14)	0.001
Age (years)		
<60	1.06 (1.03, 1.10)	0.003
>=60	1.07 (1.03, 1.11)	0.013
BMI (kg/m^2^)		
<24	1.11 (1.04, 1.17)	0.009
>=24	1.05 (1.03, 1.08)	0.001
PRA (ng/mL/h)		
PRA<=0.5	1.07 (1.03, 1.11)	<0.001
PRA>0.5	1.06 (1.05, 1.08)	<0.001
ARR		
ARR<20	1.06 (1.05, 1.09)	<0.001
ARR>=20	1.07 (1.04, 1.09)	<0.001
Current smoking		
No	1.05 (1.02, 1.08)	0.003
Yes	1.14 (1.06, 1.23)	0.006
Current drinking		
No	1.05 (1.03, 1.08)	<0.001
Yes	1.16 (1.05, 1.28)	0.003
DM		
No	1.06 (1.03, 1.09)	0.002
Yes	1.07 (1.02, 1.12)	0.039

Age, sex, BMI, smoking status, drinking status, PA, DM, CHD, cancer, ALT, AST, Cr, TC, TG, HDL-C, LDL-C, ALP, TSH, FPG, serum potassium, serum calcium, serum phosphorus, serum sodium, 24-h urinary potassium, 24-h urinary calcium,24-h urinary phosphorus, 24-h urinary sodium, PTH, 25-hydroxyvitamin D, statins, aspirin, diuretics, beta-blockers, calcium channel blockers, ACEIs/ARBs, oral hypoglycemic agents, and insulin were adjusted.

PAC, plasma aldosterone concentration; PRA, Plasma renin activity; ARR, Aldosterone-renin ratio; OR, odds ratio; CI, confidence interval.

Other abbreviations, see **Table 1**.

### 3.7 Mediation analysis for associations of PAC with osteoporosis

**Figure 5:**
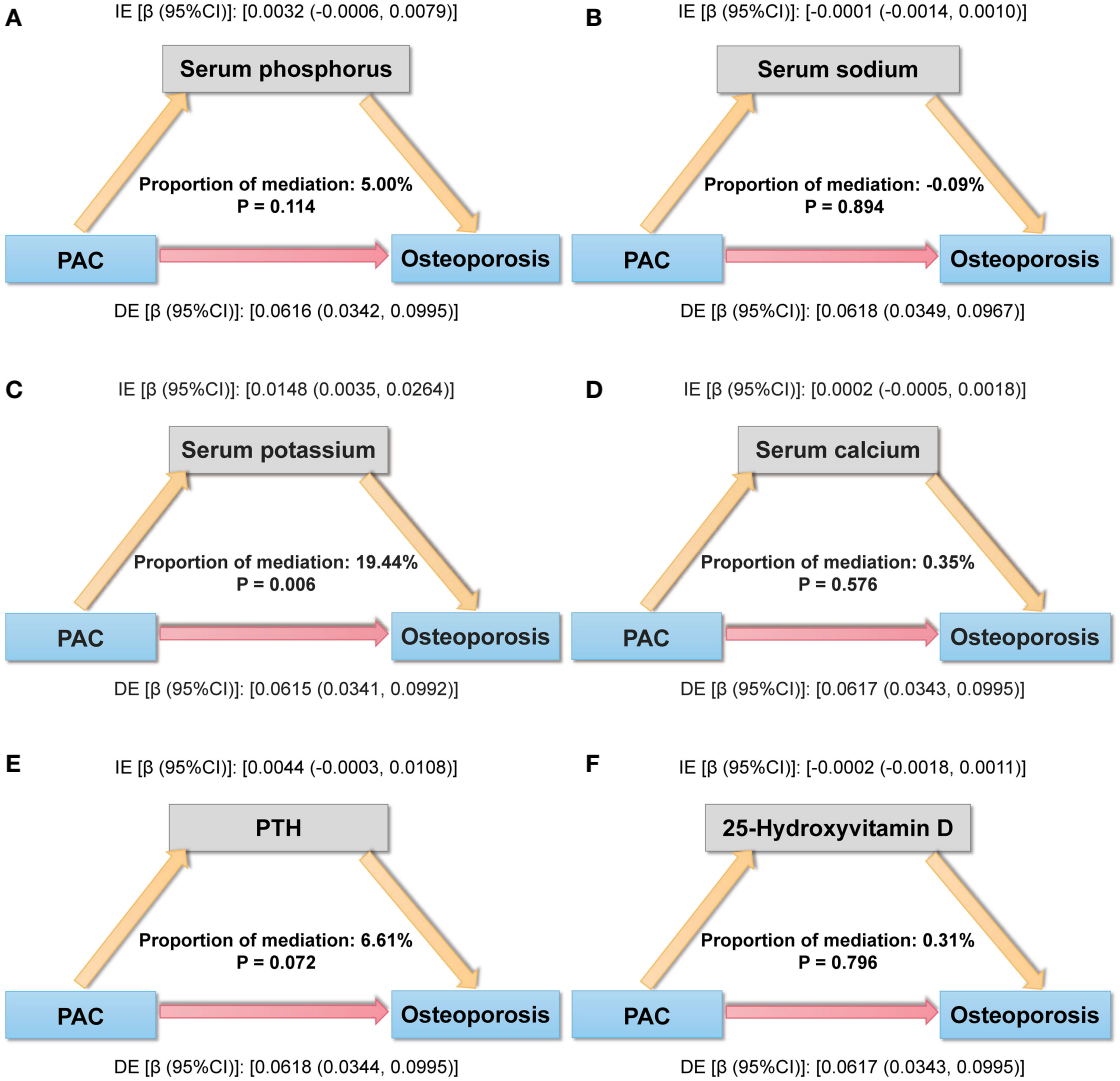
Mediation analysis model of the relationship between PAC and osteoporosis. **(A)** Serum phosphorus, **(B)** Serum sodium, **(C)** Serum potassium, **(D)** Serum calcium, **(E)** PTH, and **(F)** 25-Hydroxyvitamin D. IE, indirect effect; DE, direct effect; mediation proportion = IE/(DE +IE).

For mediation analysis, a statistically significant association between exposure, mediation, and outcome should be satisfied. **Figure 5** shows the results of a mediating analysis to explore potential mediating factors associated with PAC and osteoporosis. In the relationship between PAC and osteoporosis, we found that serum potassium was the main mediator among the six selected indicators, accounting for 19.44% of the mediated effect (P = 0.006).

## 4 Discussion

In the study, we reported that PAC was negatively associated with BMD at various sites in middle-aged and elderly hypertensive patients, that this relationship remained significant after adjusting for multiple covariates, and that high aldosterone levels raised the risk of osteoporosis. Similarly, in relation to FRAX scores, we also found a significant positive association between PAC levels and fracture risk over the next ten years. Furthermore, in mediation analyses, we further found that serum potassium played a role in the relationship between PAC and osteoporosis. Therefore, these results suggest that PAC may be a key risk factor for bone health in middle-aged and elderly hypertensive patients, increasing the risk of bone loss, osteoporosis, and future fractures.

Several previous studies have also found that aldosterone may affect calcium metabolism and influence mineral homeostasis (15, 16, 29, 30). For example, in one study, it was found that overproduction of aldosterone caused mutations in the receptors of the relevant ion channels, leading to disturbances in calcium metabolism, which in turn can directly influence steroidogenesis (15). There is also evidence of a bidirectional interaction between aldosterone and PTH, and this bidirectional interaction may lead to an increased rate of metabolic and bone disease (29). At the same time, some animal studies have further found that high PAC is associated with hypercalcemia, resulting in bone calcium loss, but treatment with the aldosterone receptor antagonist spironolactone can reverse this bone loss (30). In addition, in recent years, more and more studies have begun to find that patients with PA are at a significantly increased risk for decreased bone mass and osteoporosis (21, 22, 31–33). In 11 patients with PA and 15 matched non-PA, Salcuni AS et al. found that the PA group had a lower BMD and a higher prevalence of osteoporosis compared to the non-PA group (22). Notsu M et al. observed that the incidence and severity of vertebral fractures (VF) were significantly increased in patients with PA and that PA was an independent risk factor for VF (21). At the same time, Kim BJ et al. demonstrated that aldosterone causes deterioration of bone quality by affecting the microstructure of bone, which in turn causes osteoporosis and fractures (33). However, the above studies all have certain limitations, such as only occurring in animal experiments, small samples, the selected population being relatively limited, and relatively few variables to adjust for. In contrast, this study avoids these shortcomings. As expected, the present study not only demonstrated the relationship between PAC and BMD, but also the potential impact of PAC on osteoporosis and future fracture occurrence.

In addition, among the mediating effects, we observed that serum potassium played an important mediating effect in the development of osteoporosis prompted by PAC. Aldosterone, as a crucial mineralocorticoid, plays a pivotal regulatory role in maintaining normal blood potassium ion concentrations (34). Previous epidemiological studies have revealed the significant importance of serum potassium in maintaining skeletal health (35–37). The EPIC-Norfolk study found a correlation between the UK population’s dietary potassium intake and quantitative ultrasound assessments of BMD, along with a reduced risk of osteoporosis and fractures (36). A study conducted in Korea also found that higher potassium intake was associated with increased Total and Neck BMD, indicating the beneficial effects of dietary potassium intake on skeletal health (35). A 4-year longitudinal study on men reportedly discovered that a higher baseline consumption of potassium was linked to a lower probability of femur BMD loss in the future (37). Potassium’s beneficial impact on bone health may be attributed to the following mechanisms. Firstly, potassium contributes to the maintenance of calcium homeostasis, and potassium deprivation can lead to increased urinary calcium excretion, resulting in bone calcium loss (38). Secondly, potassium is implicated in bone regeneration, and restricted potassium channel activity may inhibit the differentiation of endothelial progenitor cells, which have the function of stimulating osteoblast differentiation and bone formation (39, 40). Additionally, bone mineral surfaces contain high levels of potassium, which is regulated by the bioactive periosteum to effectively separate minerals from the extracellular fluid. When bone cells die, excess potassium is rapidly released from the bone mineral surface, allowing a significant influx of calcium to prevent further bone loss (41–43). In conclusion, the discovery of these mediating factors may provide a basis for future mechanism elucidation.

Potential mechanisms linking PAC and BMD remain uncertain. Based on relevant research, several possible factors may explain this phenomenon. First, this may be related to calcium loss. Aldosterone increases urinary calcium excretion, leading to a calcium metabolism disorder, thereby stimulating PTH secretion, and consequently causing bone loss (31, 44). Second, inflammation may play an important role. Overabundance of aldosterone causes the release of inflammatory mediators, including interleukin-6 and tumor necrosis factor-alpha, which cause osteoclast development to be stimulated and bone resorption to be accelerated (45–47). Third, oxidative stress is involved in the negative relationship between PAC and BMD and plays an important role in bone reconstruction. It can increase the expression of bone metabolism genes and decrease the differentiation and activity of osteoblasts, thereby inducing bone loss (20, 48, 49). Finally, another potential factor to consider is the activation of the renin-angiotensin-aldosterone system (RAAS). Research has shown that RAAS and the salt corticosteroid receptor are present in human bone tissue, and high levels of angiotensin II and aldosterone may contribute to higher bone turnover and lower bone mineral density (50, 51).

There are several strengths in this study to highlight. First, to our knowledge, this study is the first to report the relationship between PAC and BMD, osteoporosis, and the risk of fracture over the next decade in middle-aged and elderly hypertensive patients. Second, compared with previous studies, this study has a larger sample size and sufficient clinical information. Third, these associations remained stable even after multivariable adjustments and sensitivity analyses. Finally, we also performed mediation analyses to explore potential mediating factors. Despite these strengths, several potential limitations should be considered when interpreting our findings. First, due to the cross-sectional nature of the study design, we were unable to determine a causal relationship between PAC and BMD. Second, we did not collect data on genetic predisposition, diet, physical activity, or sun exposure habits, which were important to bone metabolism. Thirdly, this study was performed in China, so it is uncertain whether our findings are generalizable to other countries and ethnicities. Finally, although rigorous adjusting for potential confounders is necessary, the possibility of residual confounders cannot be ruled out.

## 5 Conclusion

In conclusion, our study demonstrates that PAC is inversely associated with BMD in middle-aged and elderly hypertensive patients and that increased PAC levels increase the risk of osteoporosis and future fractures. Further mediation analyses demonstrated that serum potassium had a mediating effect on the relationship between PAC and osteoporosis. Further longitudinal studies are required to confirm our preliminary findings and elucidate underlying biological mechanisms.

## Data availability statement

The raw data supporting the conclusions of this article will be made available by the authors, without undue reservation.

## Ethics statement

This study was approved by the Research Ethics Committee of Xinjiang Uygur Autonomous Region People’s Hospital (KY2022080905). The studies were conducted in accordance with the local legislation and institutional requirements. The participants provided their written informed consent to participate in this study.

## Author contributions

SS: Data curation, Formal analysis, Investigation, Methodology, Software, Writing – original draft, Writing – review & editing. XC: Conceptualization, Data curation, Formal analysis, Methodology, Software, Writing – review & editing, Writing – original draft. JHu: Conceptualization, Formal analysis, Investigation, Methodology, Software, Writing – review & editing. QZ: Conceptualization, Data curation, Writing – review & editing. DS: Data curation, Software, Writing – review & editing. MH: Conceptualization, Supervision, Writing – review & editing. WY: Formal analysis, Software, Writing – review & editing. JHo: Methodology, Supervision, Writing – review & editing. NL: Conceptualization, Data curation, Formal analysis, Investigation, Methodology, Project administration, Supervision, Writing – review & editing.
