# Supplementary material for: Correlation between plasma aldosterone concentration and bone mineral density in middle-aged and elderly hypertensive patients: potential impact on osteoporosis and future fracture risk
Source: Front Endocrinol (Lausanne). 2024 May 14;15:1373862. doi: 10.3389/fendo.2024.1373862 (PMC11130431; doi:10.3389/fendo.2024.1373862)
Supplement: Supplementary file 1 [file DataSheet_1.docx]

Supplementary Material

# Supplemental material and methods

**Baseline examination**

Anthropometric measurements were taken by trained nurses. Data for height and weight were 3 acquired following a protocol standardized to an accuracy of 0.1 kg and 0.1 cm, respectively. Current smokers were defined as having smoked 100 cigarettes in their lifetime and currently smoking. Alcohol consumption was evaluated with questions regarding the types of alcoholic beverages, the frequency of alcohol consumption per week, and the usual amount consumed per occasion. Subjects who reported alcohol consumption >140 g/week for men and >70 g/week for women were deemed to have excessive alcohol consumption (1). Blood pressure was measured using a mercury sphygmomanometer after the patient had rested quietly for at least 10 minutes, and the average of multiple measurements was taken as the systolic and diastolic blood pressure values. All biochemical tests were measured by blood sampling after an overnight fast. For the measurement of plasma aldosterone concentrations (PAC), participants were asked to sit for 30 minutes after being active for at least 2 hours before blood collection, which took place between 8:00 and 11:00 AM.

**Definitions**

Criteria for hypertension included self-reported hypertension, current use of anti-hypertensive medication, or systolic blood pressure (SBP) ≥ 140 mmHg and/or diastolic blood pressure (DBP) ≥ 90 mmHg recorded for at least three consecutive readings. diabetes was defined as fasting serum glucose ≥7.0 mmol/L, the 2-h serum glucose of the oral glucose tolerance test ≥11.1 mmol/L, or the current use of hypoglycaemic medication or insulin. coronary heart disease (CHD) was defined as a fatal or nonfatal myocardial infarction, unstable angina, and coronary revascularization. To identify primary aldosteronism (PA) according to the Endocrine Society's Clinical Practice Guidelines, PAC was measured using radio-immunoassay (DSL-8600 ACTIVE Aldosterone Coated Tube Radioimmunoassay Kit; Diagnostic Systems Laboratories, Webster, TX). PRA was also measured by radioimmunoassay using commercial kits (Center of Beifang Biology Technique, Beijing, China). The plasma samples were divided into 2 parts: one for the determination of plasma AIa (angiotensin I) con-centrations after reacting the sample with direct antibodies and the other for the determination of plasma AIb concentrations following a 1-hour incubation at 37 °C and then reacting the sample with direct antibodies. PRA was calculated using the following formula: [AIb-AIa]/h.31 If the case was suspected of primary aldosteronism (PA; ie, PAC ≥12 ng/dL and aldosterone-renin ratio ≥20), the saline infusion test (SIT) was performed to confirm PA. The diagnosis of PA was based on SIT criteria in accordance with the Endocrine Society Guideline. Post-SIT PAC >10 ng/dL can be confirmed as PA, while post-SIT PAC 5 to 10 ng/dL can be interpreted as undetermined PA that was diagnosed or rejected based on clinical manifestations such as drug-resistant hypertension, spontaneous or diuretic-induced hypokalemia, and adrenal incidentaloma or hyperplasia found by thin layer CT scan, and post-SIT PAC <5 ng/dL makes a diagnosis of PA unlikely (2, 3). Menopausal status was defined according to World Health Organization recommendations, which regard amenorrhea lasting 12 months as indicative of the menopause (4). Cancer was based on self-report of physician diagnosis of cancer or malignancy and/or use of anticancer medications. The body mass index (BMI) was calculated as per the formula: Weight (kg)/Height^2^ (m).

**Bone Mineral Density Assessment**

Dual-energy X-ray absorptiometry (DXA) scans were performed by two health technologists who were certified radiology technologists using the bone densitometer (Horizon Wi S/N302999M, Hologic, MA, USA) (5). The DXA machine was calibrated daily with a phantom. Lumbar spine (L1–L4) and femoral regions (total femur; femur neck; Ward's triangle) scans of each subject were performed and analyzed upon test completion, with each scan taking approximately 5 min (6). The short-term in vivo coefficient of variation for the DXA machine was 1.8% for the lumbar spine and 1.2% for the femoral regions. The lumbar and femoral regions BMD were computed automatically by the DXA scanner.

**Details of the statistical analyses**.

Variables of baseline characteristics are shown as n (%) if categorical, mean (SD) if normally distributed, and median (interquartile range) if nonnormally distributed. To compare the characteristics among different PAC groups, the chi-square test was performed for categorical variables, and one-way analysis of variance, or the Kruskal-Wallis test, was performed for continuous variables with normal and skewed distributions.

Prior to any regression analysis, we tested multicollinearity among the predictors by checking for the variance inflation factor. Multicollinearity analyses revealed that variance inflation factors were smaller than 10 for all predictor variables, confirming that regression models were not affected by the presence of multicollinearity. Multivariable linear regression models were used to estimate the associations between PAC and BMD and FRAX scores, respectively. The association between PAC and osteoporosis was tested with multivariable logistic regression models. This study set five different models (Model 1: unadjusted; Model 2: adjusted for age, sex, BMI, smoking status, and drinking status; Model 3: Model 2 plus adjustment for PA, DM, CHD, and cancer. Model 4: Model 3 plus adjustment for ALT, AST, Cr, TC, TG, HDL-C, LDL-C, ALP, TSH, FPG, serum potassium, serum calcium, serum phosphorus, serum sodium, 24-h urinary potassium, 24-h urinary calcium,24-h urinary phosphorus, 24-h urinary sodium, PTH, and 25-hydroxyvitamin D. Model 5: Model 4 plus adjustment for use of statins, aspirin, diuretics, beta-blockers, calcium channel blockers, ACEIs/ARBs, oral hypoglycemic agents, and insulin.) to adjust for covariates considering that the condition of over-adjustment might exist when a large number of factors are adjusted for simultaneously. Tests for trend were conducted, assigning the median value within each tertile to the corresponding tertile. In addition, the restricted inverse square spline (four nodes at the 5th, 35th, 65th, and 95th percentiles of the PAC distribution) was used to evaluate the nonlinear relationships, and the turning points were further calculated using a recursive algorithm. Moreover, based on the turning point, we conducted a two-stage analysis before and after the turning point. Finally, subgroup analysis was performed stratified by sex, age BMI, smoking status, drinking status, and diabetes. Based on the current obesity guidelines for Chinese individuals, the BMI of 24 or higher classifies a person as overweight (7-9). Consequently, we selected this BMI threshold for our stratification process. This approach aims to thoroughly examine the impact of PAC on BMD and osteoporosis in participants in different weight categories.

Once the association between PAC and osteoporosis had been established, mediation models were constructed to examine whether the association between PAC and osteoporosis was respectively mediated by serum ions, PTH, and 25-hydroxyvitamin D. The causal steps approach, based on the influential work proposed by Baron and Kenny, was conducted to investigate the effect of PAC on the incidence of osteoporosis, partially mediated through serum ions, PTH, and 25-hydroxyvitamin D. To perform mediation analysis, it is necessary to test three pathways: Step 1, the association of PAC with osteoporosis; Step 2, the association of PAC with serum ions, PTH, and 25-hydroxyvitamin D; and Step 3, the association of serum ions, PTH, or 25-hydroxyvitamin D with osteoporosis, controlling for osteoporosis. All pathways were tested using the generalized structural equation modeling technique. Bootstrapping methods were used to estimate the 95% CI of indirect (mediated) effects. Mediation was confirmed if the bias-corrected 95% CI for the indirect effect did not include zero.

We performed a series of sensitivity analyses to assess the robustness of our findings as follows: First, due to the skewed distribution of PAC, we further validated the relationship between PAC and BMD, FRAX scores, and osteoporosis after the PAC natural logarithm (Ln) transformation. Second, we excluded data with missing values to judge consistency with the overall trend. Third, in order to verify the reliability of the results, we analyzed the relationship between PAC and BMD and osteoporosis after excluding outliers (PAC less than -3SD or more than +3SD). Fourth, we excluded patients with serious illnesses such as cancer. Fifth, we excluded such vitamin D-deficient participants with 25-hydroxyvitamin D < 20 nmol/L. Sixth, we again excluded excessively obese participants with BMI > 30 kg/m2. Seventh, due to the older population's own reduced bone mass and increased risk of osteoporosis, for this reason, we further excluded participants with age > 75 years. Finally, we again excluded patients with PA to verify the reliability of our results.

All analyses were done using R (4.2.2). All P-values were two-sided, and P-values of <0.05 denoted statistical significance.

1. **Supplementary Tables**

**Table S1**. List of medications included in the study.

| Drug class | Drug name |
| --- | --- |
| Aspirin | Aspirin |
| Statin | Atorvastatin, fluvastatin, pitavastatin, rosuvastatin, simvastatin |
| Diuretics | Acetazolamide, amiloride, benzyl hydrochlorothiazide, bumetanide, furosemide, hydrochlorothiazide, indapamide, spironolactone |
| Beta-blocker | Atenolol, bisoprolol, carvedilol, metoprolol, propranolol |
| Calcium channel blockers | Amlodipine, diltiazem, felodipine, lercanidipine, nifedipine,  verapamil |
| Angiotensin-converting enzyme inhibitors or angiotensin receptor blockers | Azilsartan, candestartan, captopril, enalapril, fosinopril, irbesartan, losartan, olmesartan, ramipril telmisartan, valsartan |
| Oral antidiabetic agents | Metformin, glipizide, gliclazide, glimepiride, glyburide, alogliptin, linagliptin, sitagliptin, vidagliptin, saxagliptin, acarbose, nateglinide, meglitinide, repaglinde, pioglitzone,dulaglutide, exenatide, liraglutide |
| Insulin | Rapid, short, intermediate, and long-acting insulins |

**Table S2.** Covariance Diagnostics

|  | VIF |
| --- | --- |
| Age | 1.9 |
| Sex | 8 |
| BMI | 1.4 |
| SBP | 1.7 |
| DBP | 1.9 |
| Current smoking | 1.9 |
| Current drinking | 1.7 |
| PA | 1.2 |
| DM | 3.4 |
| CHD | 1.2 |
| Cancer | 1.5 |
| Menopausal | 6.8 |
| ALT | 4.5 |
| AST | 4.3 |
| Cr | 1.8 |
| TC | 5.1 |
| TG | 2.1 |
| HDL.C | 1.7 |
| LDL.C | 4.2 |
| ALP | 1.4 |
| TSH | 1.1 |
| FPG | 3 |
| Serum potassium | 1.6 |
| Serum calcium | 1.1 |
| Serum phosphorus | 1.2 |
| Serum sodium | 1.2 |
| 24-h urinary potassium | 1.5 |
| 24h-urinary calcium | 1.5 |
| 24h-urinary phosphorus | 1.4 |
| 24-h urinary sodium | 1.3 |
| PTH | 1.4 |
| 25-Hydroxyvitamin D | 1.1 |
| Statins | 1.5 |
| Aspirins | 1.5 |
| Diuretics | 1.3 |
| Beta-blockers | 1.2 |
| Calcium channel blockers | 1.1 |
| ACEIs/ARBs | 1.3 |
| Oral hypoglycemic agents | 2.5 |
| Insulin | 1.3 |

VIF = 1/(1-R2). VIF step-by-step screening method: Calculate the VIF of each variable. If the maximum VIF value is ≥ 10, remove the variable with the maximum VIF value.

VIF: variance inflation factors. For other abbreviations, see Table 1.

**Table S3.** Comparison of characteristics between osteoporotic and non-osteoporotic groups

| Osteoporosis | No | Yes | P-value |
| --- | --- | --- | --- |
| Number of subjects (n) | 1069 | 361 |  |
| Age (years) | 55.72 ± 10.93 | 59.90 ± 10.72 | <0.001 |
| Sex (%) |  |  | <0.001 |
| Female | 523 (48.92%) | 235 (65.10%) |  |
| Male | 546 (51.08%) | 126 (34.90%) |  |
| BMI (kg/m^2^) | 27.16 ± 3.74) | 25.90 ± 3.88 | <0.001 |
| SBP (mmHg) | 144.75 ± 16.87 | 145.63 ± 18.5 | 0.404 |
| DBP (mmHg) | 87.10 ± 12.29 | 85.59 ± 12.38 | 0.044 |
| Current smoking (%) | 267 (24.98%) | 64 (17.73%) | 0.005 |
| Current drinking (%) | 199 (18.62%) | 53 (14.68%) | 0.090 |
| **Medical history** |  |  |  |
| PA (%) | 218 (20.39%) | 55 (15.24%) | 0.098 |
| DM (%) | 361 (33.77%) | 141 (39.06%) | 0.069 |
| Cancer (%) | 52 (4.86%) | 23 (6.37%) | 0.267 |
| CHD (%) | 63 (5.89%) | 29 (8.03%) | 0.152 |
| Menopausal (%) | 366 (34.23%) | 204 (56.50%) | <0.001 |
| **Laboratory tests** |  |  |  |
| ALT (U/L) | 23.75 (16.00-36.00) | 20.30 (15.00-33.00) | 0.005 |
| AST (U/L) | 20.53 (16.30-26.00) | 20.00 (16.00-26.00) | 0.661 |
| Cr (umol/L) | 64.82 ± 16.8 | 62.75 ± 17.24 | 0.045 |
| TC (mmol/L) | 4.56 ± 1.10 | 4.76 ± 1.0 | 0.002 |
| TG (mmol/L) | 1.64 (1.13-2.50) | 1.58 (1.15-2.41) | 0.875 |
| HDL-C (mg/dL) | 1.10 ± 0.2 | 1.16 ± 0.2 | <0.001 |
| LDL-C (mg/dL) | 2.71 ± 0.9 | 2.79 ± 0.9 | 0.153 |
| ALP (U/L) | 79.64 (60.88-101.49) | 83.77 (67.63-107.55) | 0.012 |
| TSH (uIU/mL) | 2.16 (1.45-3.41) | 2.42 (1.49-3.57) | 0.346 |
| FPG (mmol/L) | 5.93 ± 2.1 | 5.99 ± 2.21 | 0.673 |
| PAC (ng/dl) | 14.36 ± 5.7 | 18.32 ± 8.2 | <0.001 |
| Serum potassium (mmol/L) | 3.93 ± 0.34 | 3.76 ± 0.36 | <0.001 |
| Serum calcium (mmol/L) | 2.39 ± 0.71 | 2.34 ± 0.65 | 0.188 |
| Serum phosphorus (mmol/L) | 1.16 ± 0.17 | 1.12 ± 0.18 | <0.001 |
| Serum sodium (mmol/L) | 140.82 ± 2.77 | 140.70 ± 3.35 | 0.871 |
| 24-h urinary potassium (mmol/L) | 36.07 (29.24-46.55) | 38.56 (30.39-48.66) | 0.103 |
| 24-h urinary calcium (mmol/L) | 4.54 (3.04-6.40) | 5.36 (3.82-7.20) | <0.001 |
| 24-h urinary phosphorus (mmol/L) | 17.58 (12.59-23.16) | 15.89 (11.38-21.31) | 0.003 |
| 24-h urinary sodium (mmol/L) | 138.87 (95.11-188.91) | 123.95 (84.74-165.57) | 0.001 |
| PTH (pg/ml) | 46.10 (32.35-64.10) | 56.07 (36.50-76.40) | <0.001 |
| 25-hydroxyvitamin D (nmol/L) | 20.10 (13.03-29.57) | 16.80 (10.36-28.71) | 0.004 |
| **Medication** |  |  |  |
| Statins (%) | 162 (15.15%) | 64 (17.73%) | 0.246 |
| Aspirins (%) | 133 (12.44%) | 61 (16.90%) | 0.033 |
| Diuretics (%) | 102 (9.54%) | 36 (9.97%) | 0.811 |
| Beta-blockers (%) | 160 (14.97%) | 59 (16.34%) | 0.530 |
| Calcium channel blockers (%) | 530 (49.58%) | 183 (50.69%) | 0.714 |
| ACEIs/ARBs (%) | 390 (36.48%) | 138 (38.23%) | 0.553 |
| Oral hypoglycemic agents (%) | 270 (25.26%) | 105 (29.09%) | 0.153 |
| Insulin (%) | 68 (6.36%) | 33 (9.14%) | 0.075 |
| **DXA BMD T-scores** |  |  |  |
| Lumbar 1 | -0.26 ± 1.4 | -2.55 ± 0.8 | <0.001 |
| Lumbar 2 | -0.21 ± 1.3 | -2.56 ± 0.9 | <0.001 |
| Lumbar 3 | -0.13 ± 1.4 | -2.68 ± 0.9 | <0.001 |
| Lumbar 4 | -0.03 ± 1.46 | -2.49 ± 1.0 | <0.001 |
| Neck | -0.46 ± 0.9 | -1.77 ± 0.8 | <0.001 |
| Wards | -0.71 ± 1.1 | -2.12 ± 1.0 | <0.001 |
| Total | 0.20 ± 0.9 | -1.04 ± 0.95 | <0.001 |
| **FRAX scores (%)** |  |  |  |
| MOF | 2.68 ± 1.3 | 7.11 ± 3.9 | <0.001 |
| HF | 0.71 ± 0.9 | 3.90 ± 3.5 | <0.001 |

Data are presented as mean ± standard deviation, median (interquartile range), or as numbers, and percentages.

Abbreviations: BMI, body mass index; SBP, systolic blood pressure; DBP, diastolic blood pressure; PA, primary aldosteronism; DM, diabetes mellitus; CHD, coronary heart disease; ALT, alanine transaminase; AST, aspartate transaminase; Cr, creatinine; TC, total cholesterol; TG, triglyceride; HDL-C, high-density lipoprotein cholesterol; LDL-C, low-density lipoprotein cholesterol; ALP, alkaline phosphatase; TSH, thyroid stimulating hormone; FPG, fasting plasma glucose; PTH, parathyroid hormone; PAC, plasma aldosterone concentration; ARBs, angiotensin receptor blockers; ACEIs, angiotensin-converting enzyme inhibitors; BMD, bone mineral density; Neck, neck of the femur; Wards, Ward's triangle; Total, total femur; MOF, major osteoporotic fracture; HF, hip fracture.

**Table S4.** Among women, we further stratified our analyses according to menopausal status.

| Menopausal | No (188) | Yes (570) |
| --- | --- | --- |
|  | β / OR (%95CI) P value | |
| Lumbar 1 | -0.06 (-0.10, -0.03) <0.001 | -0.04 (-0.06, -0.02) <0.001 |
| Lumbar 2 | -0.05 (-0.09, -0.02) 0.006 | -0.05 (-0.08, -0.03) <0.001 |
| Lumbar 3 | -0.05 (-0.09, -0.01) 0.013 | -0.05 (-0.08, -0.03) <0.001 |
| Lumbar 4 | -0.05 (-0.09, -0.01) 0.018 | -0.05 (-0.08, -0.03) <0.001 |
| Neck | -0.05 (-0.08, -0.03) <0.001 | -0.05 (-0.07, -0.03) <0.001 |
| Wards | -0.06 (-0.09, -0.04) <0.001 | -0.05 (-0.07, -0.03) <0.001 |
| Toal | -0.06 (-0.09, -0.04) <0.001 | -0.05 (-0.07, -0.04) <0.001 |
| MOF | 0.04 (0.01, 0.07) 0.007 | 0.14 (0.10, 0.19) <0.001 |
| HF | 0.03 (0.07, 0.05) 0.007 | 0.11 (0.08, 0.14) <0.001 |
| Osteoporosis | 1.09 (1.00, 1.20) 0.055 | 1.05 (1.01, 1.10) 0.018 |

Age, sex, BMI, smoking status, drinking status, PA, DM, CHD, cancer, ALT, AST, Cr, TC, TG, HDL-C, LDL-C, ALP, TSH, FPG, serum potassium, serum calcium, serum phosphorus, serum sodium, 24-h urinary potassium, 24-h urinary calcium,24-h urinary phosphorus, 24-h urinary sodium, PTH, 25-hydroxyvitamin D, statins, aspirin, diuretics, beta-blockers, calcium channel blockers, ACEIs/ARBs, oral hypoglycemic agents, and insulin were adjusted.

Abbreviations: Neck, neck of the femur; Wards, Ward's triangle; MOF, major osteoporotic fracture; HF, hip fracture; β, regression coefficient; OR, odds ratio; CI, confidence interval.

Other abbreviations, see Table 1.

**Table S5.** PAC after Ln transformation in relation to BMD.

| Exposure | Model 1 | Model 2 | Model 3 | Model 4 | Model 5 |
| --- | --- | --- | --- | --- | --- |
|  | β (95% CI) | β (95% CI) | β (95% CI) | β (95% CI) | β (95% CI) |
| **Lumbar 1** |  |  |  |  |  |
| ln PAC (per 1-unit increase) | -1.54 (-1.76, -1.32) | -1.43 (-1.65, -1.22) | -1.26 (-1.52, -1.00) | -1.31 (-1.57, -1.04) | -1.31 (-1.58, -1.05) |
| ln PAC (per 1-SD increase) | -0.56 (-0.64, -0.48) | -0.52 (-0.60, -0.44) | -0.46 (-0.55, -0.36) | -0.48 (-0.57, -0.38) | -0.48 (-0.57, -0.38) |
| **Lumbar 2** |  |  |  |  |  |
| ln PAC (per 1-unit increase) | -1.53 (-1.75, -1.31) | -1.42 (-1.64, -1.21) | -1.26 (-1.52, -1.00) | -1.32 (-1.58, -1.05) | -1.32 (-1.59, -1.06) |
| ln PAC (per 1-SD increase) | -0.55 (-0.64, -0.47) | -0.52 (-0.59, -0.44) | -0.46 (-0.55, -0.36) | -0.48 (-0.57, -0.38) | -0.48 (-0.58, -0.38) |
| **Lumbar 3** |  |  |  |  |  |
| ln PAC (per 1-unit increase) | -1.63 (-1.86, -1.40) | -1.51 (-1.73, -1.29) | -1.26 (-1.53, -0.99) | -1.33 (-1.61, -1.06) | -1.36 (-1.63, -1.08) |
| ln PAC (per 1-SD increase) | -0.59 (-0.68, -0.51) | -0.55 (-0.63, -0.47) | -0.46 (-0.56, -0.36) | -0.48 (-0.58, -0.38) | -0.49 (-0.59, -0.39) |
| **Lumbar 4** |  |  |  |  |  |
| ln PAC (per 1-unit increase) | -1.77 (-2.00, -1.53) | -1.65 (-1.87, -1.42) | -1.39 (-1.67, -1.12) | -1.45 (-1.73, -1.17) | -1.46 (-1.74, -1.18) |
| ln PAC (per 1-SD increase) | -0.64 (-0.73, -0.56) | -0.60 (-0.68, -0.52) | -0.51 (-0.60, -0.41) | -0.53 (-0.63, -0.43) | -0.53 (-0.63, -0.43) |
| **Neck** |  |  |  |  |  |
| ln PAC (per 1-unit increase) | -1.25 (-1.40, -1.11) | -1.25 (-1.38, -1.11) | -1.07 (-1.23, -0.90) | -1.07 (-1.24, -0.91) | -1.07 (-1.24, -0.90) |
| ln PAC (per 1-SD increase) | -0.46 (-0.51, -0.40) | -0.45 (-0.50, -0.40) | -0.39 (-0.45, -0.33) | -0.39 (-0.45, -0.33) | -0.39 (-0.45, -0.33) |
| **Wards** |  |  |  |  |  |
| ln PAC (per 1-unit increase) | -1.42 (-1.58, -1.25) | -1.44 (-1.59, -1.28) | -1.23 (-1.42, -1.04) | -1.22 (-1.41, -1.03) | -1.22 (-1.41, -1.03) |
| ln PAC (per 1-SD increase) | -0.51 (-0.57, -0.46) | -0.52 (-0.58, -0.47) | -0.45 (-0.51, -0.38) | -0.44 (-0.51, -0.37) | -0.44 (-0.51, -0.37) |
| **Total** |  |  |  |  |  |
| ln PAC (per 1-unit increase) | -1.50 (-1.63, -1.36) | -1.46 (-1.59, -1.34) | -1.26 (-1.41, -1.10) | -1.25 (-1.41, -1.10) | -1.26 (-1.42, -1.11) |
| ln PAC (per 1-SD increase) | -0.54 (-0.59, -0.49) | -0.53 (-0.58, -0.49) | -0.46 (-0.51, -0.40) | -0.45 (-0.51, -0.40) | -0.46 (-0.51, -0.40) |

Model 1: no covariates were adjusted.

Model 2: age, sex, BMI, smoking status, and drinking status were adjusted.

Model 3: Model 2 plus adjustment for PA, DM, CHD, and cancer.

Model 4: Model 3 plus adjustment for ALT, AST, Cr, TC, TG, HDL-C, LDL-C, ALP, TSH, FPG, serum potassium, serum calcium, serum phosphorus, serum sodium, 24-h urinary potassium, 24-h urinary calcium,24-h urinary phosphorus, 24-h urinary sodium, PTH, and 25-hydroxyvitamin D.

Model 5: Model 4 plus adjustment for use of statins, aspirin, diuretics, beta-blockers, calcium channel blockers, ACEIs/ARBs, oral hypoglycemic agents, and insulin.

Abbreviations: PAC, plasma aldosterone concentration; BMD, bone mineral density; Neck, neck of the femur; Wards, Ward's triangle; β, regression coefficient; CI, confidence interval.

Other abbreviations, see Table 1.

**Table S6.** PAC after Ln transformation in relation to FRAX scores.

| Exposure | Model 1 | Model 2 | Model 3 | Model 4 | Model 5 |
| --- | --- | --- | --- | --- | --- |
|  | β (95% CI) | β (95% CI) | β (95% CI) | β (95% CI) | β (95% CI) |
| **MOF** |  |  |  |  |  |
| ln PAC (per 1-unit increase) | 3.19 (2.79, 3.58) | 3.11 (2.75, 3.48) | 2.51 (2.06, 2.95) | 2.59 (2.14, 3.04) | 2.60 (2.15, 3.05) |
| ln PAC (per 1-SD increase) | 1.16 (1.01, 1.30) | 1.13 (1.00, 1.26) | 0.91 (0.75, 1.07) | 0.94 (0.78, 1.10) | 0.94 (0.78, 1.11) |
| **HF** |  |  |  |  |  |
| ln PAC (per 1-unit increase) | 2.40 (2.09, 2.72) | 2.42 (2.11, 2.72) | 1.99 (1.61, 2.36) | 2.06 (1.69, 2.44) | 2.07 (1.69, 2.45) |
| ln PAC (per 1-SD increase) | 0.87 (0.76, 0.99) | 0.88 (0.77, 0.99) | 0.72 (0.59, 0.86) | 0.75 (0.61, 0.89) | 0.75 (0.61, 0.89) |

Model 1: no covariates were adjusted.

Model 2: age, sex, BMI, smoking status, and drinking status were adjusted.

Model 3: Model 2 plus adjustment for PA, DM, CHD, and cancer.

Model 4: Model 3 plus adjustment for ALT, AST, Cr, TC, TG, HDL-C, LDL-C, ALP, TSH, FPG, serum potassium, serum calcium, serum phosphorus, serum sodium, 24-h urinary potassium, 24-h urinary calcium,24-h urinary phosphorus, 24-h urinary sodium, PTH, and 25-hydroxyvitamin D.

Model 5: Model 4 plus adjustment for use of statins, aspirin, diuretics, beta-blockers, calcium channel blockers, ACEIs/ARBs, oral hypoglycemic agents, and insulin.

Abbreviations: PAC, plasma aldosterone concentration; MOF, major osteoporotic fracture; HF, hip fracture; β, regression coefficient; CI, confidence interval.

Other abbreviations, see Table 1.

**Table S7.** PAC after Ln transformation in relation to osteoporosis.

| Exposure | Model 1 | Model 2 | Model 3 | Model 4 | Model 5 |
| --- | --- | --- | --- | --- | --- |
|  | OR (95% CI) | OR (95% CI) | OR (95% CI) | OR (95% CI) | OR (95% CI) |
| **Osteoporosis** |  |  |  |  |  |
| ln PAC (per 1-unit increase) | 5.91 (4.13, 8.47) | 5.85 (4.04, 8.48) | 3.85 (2.46, 6.02) | 4.12 (2.59, 6.54) | 4.14 (2.59, 6.60) |
| ln PAC (per 1-SD increase) | 1.91 (1.67, 2.17) | 1.90 (1.66, 2.17) | 1.63 (1.39, 1.92) | 1.67 (1.41, 1.98) | 1.67 (1.41, 1.98) |

Model 1: no covariates were adjusted.

Model 2: age, sex, BMI, smoking status, and drinking status were adjusted.

Model 3: Model 2 plus adjustment for PA, DM, CHD, and cancer.

Model 4: Model 3 plus adjustment for ALT, AST, Cr, TC, TG, HDL-C, LDL-C, ALP, TSH, FPG, serum potassium, serum calcium, serum phosphorus, serum sodium, 24-h urinary potassium, 24-h urinary calcium,24-h urinary phosphorus, 24-h urinary sodium, PTH, and 25-hydroxyvitamin D.

Model 5: Model 4 plus adjustment for use of statins, aspirin, diuretics, beta-blockers, calcium channel blockers, ACEIs/ARBs, oral hypoglycemic agents, and insulin.

Abbreviations: PAC, plasma aldosterone concentration; OR, odds ratio; CI, confidence interval.

Other abbreviations, see Table 1.

**Table S8.** Sensitivity analyses of the association of PAC with BMD, FRAX scores, and osteoporosis using a no-missing value dataset.

|  | Model 1 | Model 2 | Model 3 | Model 4 | Model 5 |
| --- | --- | --- | --- | --- | --- |
|  | β / OR (95% CI) | β / OR (95% CI) | β / OR (95% CI) | β / OR (95% CI) | β / OR (95% CI) |
| **Lumbar 1** |  |  |  |  |  |
| PAC (per 1-ng/dL increase) | -0.07 (-0.09, -0.06) | -0.05 (-0.07, -0.04) | -0.05 (-0.07, -0.03) | -0.05 (-0.07, -0.04) | -0.05 (-0.07, -0.03) |
| Tertiles of PAC |  |  |  |  |  |
| Tertile 1 | Reference | Reference | Reference | Reference | Reference |
| Tertile 2 | -0.39 (-0.59, -0.18) | -0.40 (-0.60, -0.20) | -0.40 (-0.60, -0.20) | -0.40 (-0.60, -0.20) | -0.40 (-0.60, -0.20) |
| Tertile 3 | -1.39 (-1.60, -1.18) | -1.09 (-1.33, -0.84) | -1.07 (-1.31, -0.83) | -1.09 (-1.33, -0.84) | -1.07 (-1.31, -0.83) |
| P for trend | <0.001 | <0.001 | <0.001 | <0.001 | <0.001 |
| **Lumbar 2** |  |  |  |  |  |
| PAC (per 1-ng/dL increase) | -0.07 (-0.08, -0.05) | -0.05 (-0.06, -0.03) | -0.04 (-0.06, -0.03) | -0.05 (-0.06, -0.03) | -0.04 (-0.06, -0.03) |
| Tertiles of PAC |  |  |  |  |  |
| Tertile 1 | Reference | Reference | Reference | Reference | Reference |
| Tertile 2 | -0.45 (-0.66, -0.24) | -0.46 (-0.66, -0.25) | -0.46 (-0.67, -0.26) | -0.46 (-0.66, -0.25) | -0.46 (-0.67, -0.26) |
| Tertile 3 | -1.38 (-1.60, -1.17) | -1.06 (-1.31, -0.81) | -1.06 (-1.31, -0.81) | -1.06 (-1.31, -0.81) | -1.06 (-1.31, -0.81) |
| P for trend | <0.001 | <0.001 | <0.001 | <0.001 | <0.001 |
| **Lumbar 3** |  |  |  |  |  |
| PAC (per 1-ng/dL increase) | -0.07 (-0.09, -0.06) | -0.05 (-0.06, -0.03) | -0.05 (-0.06, -0.03) | -0.05 (-0.06, -0.03) | -0.05 (-0.06, -0.03) |
| Tertiles of PAC |  |  |  |  |  |
| Tertile 1 | Reference | Reference | Reference | Reference | Reference |
| Tertile 2 | -0.45 (-0.67, -0.23) | -0.44 (-0.66, -0.23) | -0.45 (-0.67, -0.24) | -0.44 (-0.66, -0.23) | -0.45 (-0.67, -0.24) |
| Tertile 3 | -1.49 (-1.72, -1.27) | -1.09 (-1.35, -0.84) | -1.10 (-1.36, -0.84) | -1.09 (-1.35, -0.84) | -1.10 (-1.36, -0.84) |
| P for trend | <0.001 | <0.001 | <0.001 | <0.001 | <0.001 |
| **Lumbar 4** |  |  |  |  |  |
| PAC (per 1-ng/dL increase) | -0.09 (-0.10, -0.07) | -0.06 (-0.08, -0.05) | -0.06 (-0.08, -0.05) | -0.06 (-0.08, -0.05) | -0.06 (-0.08, -0.05) |
| Tertiles of PAC |  |  |  |  |  |
| Tertile 1 | Reference | Reference | Reference | Reference | Reference |
| Tertile 2 | -0.49 (-0.71, -0.26) | -0.48 (-0.70, -0.26) | -0.47 (-0.69, -0.26) | -0.48 (-0.70, -0.26) | -0.47 (-0.69, -0.26) |
| Tertile 3 | -1.59 (-1.82, -1.37) | -1.18 (-1.44, -0.91) | -1.17 (-1.44, -0.90) | -1.18 (-1.44, -0.91) | -1.17 (-1.44, -0.90) |
| P for trend | <0.001 | <0.001 | <0.001 | <0.001 | <0.001 |
| **Neck** |  |  |  |  |  |
| PAC (per 1-ng/dL increase) | -0.06 (-0.07, -0.05) | -0.04 (-0.05, -0.03) | -0.04 (-0.05, -0.03) | -0.04 (-0.05, -0.03) | -0.04 (-0.05, -0.03) |
| Tertiles of PAC |  |  |  |  |  |
| Tertile 1 | Reference | Reference | Reference | Reference | Reference |
| Tertile 2 | -0.39 (-0.53, -0.25) | -0.38 (-0.52, -0.25) | -0.39 (-0.52, -0.26) | -0.38 (-0.52, -0.25) | -0.39 (-0.52, -0.26) |
| Tertile 3 | -1.08 (-1.22, -0.94) | -0.83 (-0.99, -0.67) | -0.82 (-0.98, -0.66) | -0.83 (-0.99, -0.67) | -0.82 (-0.98, -0.66) |
| P for trend | <0.001 | <0.001 | <0.001 | <0.001 | <0.001 |
| **Wards** |  |  |  |  |  |
| PAC (per 1-ng/dL increase) | -0.07 (-0.08, -0.06) | -0.05 (-0.06, -0.04) | -0.05 (-0.06, -0.04) | -0.05 (-0.06, -0.04) | -0.05 (-0.06, -0.04) |
| Tertiles of PAC |  |  |  |  |  |
| Tertile 1 | Reference | Reference | Reference | Reference | Reference |
| Tertile 2 | -0.41 (-0.57, -0.25) | -0.42 (-0.57, -0.27) | -0.43 (-0.58, -0.28) | -0.42 (-0.57, -0.27) | -0.43 (-0.58, -0.28) |
| Tertile 3 | -1.23 (-1.39, -1.07) | -1.01 (-1.19, -0.82) | -1.01 (-1.19, -0.82) | -1.01 (-1.19, -0.82) | -1.01 (-1.19, -0.82) |
| P for trend | <0.001 | <0.001 | <0.001 | <0.001 | <0.001 |
| **Total** |  |  |  |  |  |
| PAC (per 1-ng/dL increase) | -0.07 (-0.08, -0.07) | -0.05 (-0.06, -0.04) | -0.05 (-0.06, -0.04) | -0.05 (-0.06, -0.04) | -0.05 (-0.06, -0.04) |
| Tertiles of PAC |  |  |  |  |  |
| Tertile 1 | Reference | Reference | Reference | Reference | Reference |
| Tertile 2 | -0.50 (-0.63, -0.37) | -0.47 (-0.59, -0.35) | -0.48 (-0.60, -0.36) | -0.47 (-0.59, -0.35) | -0.48 (-0.60, -0.36) |
| Tertile 3 | -1.29 (-1.42, -1.16) | -0.99 (-1.13, -0.84) | -1.00 (-1.14, -0.85) | -0.99 (-1.13, -0.84) | -1.00 (-1.14, -0.85) |
| P for trend | <0.001 | <0.001 | <0.001 | <0.001 | <0.001 |
| **MOF** |  |  |  |  |  |
| PAC (per 1-ng/dL increase) | 0.17 (0.15, 0.20) | 0.14 (0.11, 0.16) | 0.14 (0.11, 0.16) | 0.14 (0.11, 0.16) | 0.14 (0.11, 0.16) |
| Tertiles of PAC |  |  |  |  |  |
| Tertile 1 | Reference | Reference | Reference | Reference | Reference |
| Tertile 2 | 0.38 (0.03, 0.73) | 0.33 (-0.00, 0.66) | 0.34 (0.01, 0.67) | 0.33 (-0.00, 0.66) | 0.34 (0.01, 0.67) |
| Tertile 3 | 2.58 (2.22, 2.94) | 1.82 (1.42, 2.22) | 1.84 (1.43, 2.24) | 1.82 (1.42, 2.22) | 1.84 (1.43, 2.24) |
| P for trend | <0.001 | <0.001 | <0.001 | <0.001 | <0.001 |
| **HF** |  |  |  |  |  |
| PAC (per 1-ng/dL increase) | 0.13 (0.11, 0.15) | 0.11 (0.09, 0.13) | 0.11 (0.09, 0.13) | 0.11 (0.09, 0.13) | 0.11 (0.09, 0.13) |
| Tertiles of PAC |  |  |  |  |  |
| Tertile 1 | Reference | Reference | Reference | Reference | Reference |
| Tertile 2 | 0.24 (-0.05, 0.52) | 0.17 (-0.10, 0.44) | 0.18 (-0.09, 0.45) | 0.17 (-0.10, 0.44) | 0.18 (-0.09, 0.45) |
| Tertile 3 | 1.82 (1.53, 2.10) | 1.33 (1.00, 1.66) | 1.34 (1.01, 1.68) | 1.33 (1.00, 1.66) | 1.34 (1.01, 1.68) |
| P for trend | <0.001 | <0.001 | <0.001 | <0.001 | <0.001 |
| **Osteoporosis** |  |  |  |  |  |
| PAC (per 1-ng/dL increase) | 1.09 (1.07, 1.11) | 1.06 (1.03, 1.09) | 1.06 (1.03, 1.09) | 1.06 (1.03, 1.09) | 1.06 (1.03, 1.09) |
| Tertiles of PAC |  |  |  |  |  |
| Tertile 1 | Reference | Reference | Reference | Reference | Reference |
| Tertile 2 | 1.22 (0.84, 1.77) | 1.22 (0.82, 1.80) | 1.24 (0.84, 1.85) | 1.22 (0.82, 1.80) | 1.24 (0.84, 1.85) |
| Tertile 3 | 4.37 (3.12, 6.14) | 3.19 (2.09, 4.86) | 3.24 (2.11, 4.97) | 3.19 (2.09, 4.86) | 3.24 (2.11, 4.97) |
| P for trend | <0.001 | <0.001 | <0.001 | <0.001 | <0.001 |

Model 1: no covariates were adjusted.

Model 2: age, sex, BMI, smoking status, and drinking status were adjusted.

Model 3: Model 2 plus adjustment for PA, DM, CHD, and cancer.

Model 4: Model 3 plus adjustment for ALT, AST, Cr, TC, TG, HDL-C, LDL-C, ALP, TSH, FPG, serum potassium, serum calcium, serum phosphorus, serum sodium, 24-h urinary potassium, 24-h urinary calcium,24-h urinary phosphorus, 24-h urinary sodium, PTH, and 25-hydroxyvitamin D.

Model 5: Model 4 plus adjustment for use of statins, aspirin, diuretics, beta-blockers, calcium channel blockers, ACEIs/ARBs, oral hypoglycemic agents, and insulin.

Abbreviations: PAC, plasma aldosterone concentration; BMD, bone mineral density; Neck, neck of the femur; Wards, Ward's triangle; MOF, major osteoporotic fracture; HF, hip fracture; β, regression coefficient; OR, odds ratio; CI, confidence interval.

Other abbreviations, see Table 1.

**Table S9.** Sensitivity analysis of the relationship between PAC with BMD, FRAX scores, and osteoporosis was performed after excluding outliers.

|  | Model 1 | Model 2 | Model 3 | Model 4 | Model 5 |
| --- | --- | --- | --- | --- | --- |
|  | β / OR (95% CI) | β / OR (95% CI) | β / OR (95% CI) | β / OR (95% CI) | β / OR (95% CI) |
| **Lumbar 1** |  |  |  |  |  |
| PAC (per 1-ng/dL increase) | -0.11 (-0.13, -0.10) | -0.10 (-0.12, -0.09) | -0.09 (-0.11, -0.08) | -0.10 (-0.11, -0.08) | -0.10 (-0.12, -0.08) |
| Tertiles of PAC |  |  |  |  |  |
| Tertile 1 | Reference | Reference | Reference | Reference | Reference |
| Tertile 2 | -0.31 (-0.51, -0.11) | -0.33 (-0.52, -0.14) | -0.32 (-0.51, -0.12) | -0.34 (-0.53, -0.15) | -0.34 (-0.53, -0.15) |
| Tertile 3 | -1.35 (-1.54, -1.15) | -1.24 (-1.43, -1.05) | -1.10 (-1.32, -0.87) | -1.10 (-1.33, -0.87) | -1.10 (-1.33, -0.87) |
| P for trend | <0.001 | <0.001 | <0.001 | <0.001 | <0.001 |
| **Lumbar 2** |  |  |  |  |  |
| PAC (per 1-ng/dL increase) | -0.11 (-0.13, -0.10) | -0.11 (-0.12, -0.09) | -0.10 (-0.12, -0.08) | -0.10 (-0.12, -0.08) | -0.10 (-0.12, -0.08) |
| Tertiles of PAC |  |  |  |  |  |
| Tertile 1 | Reference | Reference | Reference | Reference | Reference |
| Tertile 2 | -0.39 (-0.59, -0.19) | -0.42 (-0.61, -0.23) | -0.40 (-0.60, -0.21) | -0.43 (-0.62, -0.24) | -0.43 (-0.63, -0.24) |
| Tertile 3 | -1.37 (-1.56, -1.17) | -1.25 (-1.44, -1.06) | -1.10 (-1.33, -0.88) | -1.11 (-1.34, -0.88) | -1.11 (-1.34, -0.88) |
| P for trend | <0.001 | <0.001 | <0.001 | <0.001 | <0.001 |
| **Lumbar 3** |  |  |  |  |  |
| PAC (per 1-ng/dL increase) | -0.12 (-0.14, -0.11) | -0.11 (-0.13, -0.10) | -0.10 (-0.12, -0.08) | -0.10 (-0.12, -0.08) | -0.10 (-0.12, -0.08) |
| Tertiles of PAC |  |  |  |  |  |
| Tertile 1 | Reference | Reference | Reference | Reference | Reference |
| Tertile 2 | -0.41 (-0.61, -0.20) | -0.43 (-0.63, -0.24) | -0.40 (-0.60, -0.21) | -0.43 (-0.63, -0.24) | -0.44 (-0.64, -0.25) |
| Tertile 3 | -1.48 (-1.68, -1.27) | -1.34 (-1.54, -1.14) | -1.14 (-1.37, -0.90) | -1.15 (-1.39, -0.92) | -1.17 (-1.41, -0.93) |
| P for trend | <0.001 | <0.001 | <0.001 | <0.001 | <0.001 |
| **Lumbar 4** |  |  |  |  |  |
| PAC (per 1-ng/dL increase) | -0.13 (-0.14, -0.11) | -0.12 (-0.13, -0.10) | -0.10 (-0.12, -0.08) | -0.10 (-0.12, -0.08) | -0.10 (-0.12, -0.08) |
| Tertiles of PAC |  |  |  |  |  |
| Tertile 1 | Reference | Reference | Reference | Reference | Reference |
| Tertile 2 | -0.42 (-0.63, -0.22) | -0.44 (-0.64, -0.24) | -0.41 (-0.61, -0.21) | -0.44 (-0.64, -0.23) | -0.44 (-0.64, -0.24) |
| Tertile 3 | -1.51 (-1.72, -1.31) | -1.39 (-1.59, -1.19) | -1.17 (-1.41, -0.93) | -1.18 (-1.42, -0.94) | -1.19 (-1.43, -0.95) |
| P for trend | <0.001 | <0.001 | <0.001 | <0.001 | <0.001 |
| **Neck** |  |  |  |  |  |
| PAC (per 1-ng/dL increase) | -0.09 (-0.10, -0.08) | -0.09 (-0.10, -0.08) | -0.08 (-0.09, -0.07) | -0.08 (-0.09, -0.06) | -0.08 (-0.09, -0.06) |
| Tertiles of PAC |  |  |  |  |  |
| Tertile 1 | Reference | Reference | Reference | Reference | Reference |
| Tertile 2 | -0.38 (-0.51, -0.25) | -0.42 (-0.54, -0.30) | -0.39 (-0.51, -0.27) | -0.40 (-0.52, -0.28) | -0.40 (-0.52, -0.28) |
| Tertile 3 | -1.07 (-1.20, -0.94) | -1.06 (-1.18, -0.94) | -0.89 (-1.04, -0.75) | -0.87 (-1.02, -0.73) | -0.87 (-1.01, -0.72) |
| P for trend | <0.001 | <0.001 | <0.001 | <0.001 | <0.001 |
| **Wards** |  |  |  |  |  |
| PAC (per 1-ng/dL increase) | -0.10 (-0.11, -0.09) | -0.10 (-0.11, -0.09) | -0.09 (-0.10, -0.08) | -0.09 (-0.10, -0.07) | -0.09 (-0.10, -0.07) |
| Tertiles of PAC |  |  |  |  |  |
| Tertile 1 | Reference | Reference | Reference | Reference | Reference |
| Tertile 2 | -0.40 (-0.55, -0.25) | -0.46 (-0.60, -0.32) | -0.42 (-0.56, -0.28) | -0.44 (-0.58, -0.30) | -0.44 (-0.58, -0.30) |
| Tertile 3 | -1.21 (-1.36, -1.06) | -1.22 (-1.36, -1.08) | -1.02 (-1.19, -0.86) | -1.00 (-1.16, -0.83) | -1.00 (-1.16, -0.83) |
| P for trend | <0.001 | <0.001 | <0.001 | <0.001 | <0.001 |
| **Total** |  |  |  |  |  |
| PAC (per 1-ng/dL increase) | -0.11 (-0.12, -0.10) | -0.11 (-0.11, -0.10) | -0.09 (-0.10, -0.08) | -0.09 (-0.10, -0.08) | -0.09 (-0.10, -0.08) |
| Tertiles of PAC |  |  |  |  |  |
| Tertile 1 | Reference | Reference | Reference | Reference | Reference |
| Tertile 2 | -0.44 (-0.57, -0.32) | -0.48 (-0.60, -0.37) | -0.44 (-0.55, -0.32) | -0.45 (-0.56, -0.33) | -0.45 (-0.57, -0.34) |
| Tertile 3 | -1.27 (-1.39, -1.15) | -1.24 (-1.35, -1.12) | -1.04 (-1.17, -0.90) | -1.02 (-1.15, -0.88) | -1.02 (-1.16, -0.89) |
| P for trend | <0.001 | <0.001 | <0.001 | <0.001 | <0.001 |
| **MOF** |  |  |  |  |  |
| PAC (per 1-ng/dL increase) | 0.23 (0.21, 0.26) | 0.23 (0.20, 0.25) | 0.20 (0.17, 0.23) | 0.20 (0.17, 0.23) | 0.20 (0.17, 0.23) |
| Tertiles of PAC |  |  |  |  |  |
| Tertile 1 | Reference | Reference | Reference | Reference | Reference |
| Tertile 2 | 0.33 (-0.01, 0.67) | 0.39 (0.08, 0.71) | 0.26 (-0.05, 0.58) | 0.34 (0.03, 0.66) | 0.35 (0.03, 0.66) |
| Tertile 3 | 2.55 (2.21, 2.89) | 2.44 (2.12, 2.76) | 1.94 (1.57, 2.32) | 1.97 (1.59, 2.35) | 1.97 (1.59, 2.35) |
| P for trend | <0.001 | <0.001 | <0.001 | <0.001 | <0.001 |
| **HF** |  |  |  |  |  |
| PAC (per 1-ng/dL increase) | 0.17 (0.15, 0.20) | 0.18 (0.16, 0.20) | 0.16 (0.13, 0.19) | 0.16 (0.14, 0.19) | 0.16 (0.14, 0.19) |
| Tertiles of PAC |  |  |  |  |  |
| Tertile 1 | Reference | Reference | Reference | Reference | Reference |
| Tertile 2 | 0.20 (-0.07, 0.47) | 0.22 (-0.04, 0.48) | 0.13 (-0.14, 0.39) | 0.19 (-0.07, 0.46) | 0.20 (-0.07, 0.46) |
| Tertile 3 | 1.82 (1.55, 2.09) | 1.83 (1.56, 2.09) | 1.47 (1.15, 1.78) | 1.50 (1.18, 1.81) | 1.50 (1.18, 1.82) |
| P for trend | <0.001 | <0.001 | <0.001 | <0.001 | <0.001 |
| **Osteoporosis** |  |  |  |  |  |
| PAC (per 1-ng/dL increase) | 1.14 (1.11, 1.17) | 1.14 (1.11, 1.17) | 1.11 (1.08, 1.15) | 1.12 (1.08, 1.16) | 1.12 (1.08, 1.16) |
| Tertiles of PAC |  |  |  |  |  |
| Tertile 1 | Reference | Reference | Reference | Reference | Reference |
| Tertile 2 | 1.22 (0.85, 1.73) | 1.27 (0.88, 1.82) | 1.20 (0.83, 1.73) | 1.27 (0.87, 1.84) | 1.28 (0.88, 1.86) |
| Tertile 3 | 4.53 (3.31, 6.21) | 4.44 (3.20, 6.17) | 3.38 (2.29, 4.97) | 3.47 (2.34, 5.15) | 3.53 (2.36, 5.27) |
| P for trend | <0.001 | <0.001 | <0.001 | <0.001 | <0.001 |

Values more than 3SD from the mean were deemed as outliers, they were removed and the analysis repeated.

Model 1: no covariates were adjusted.

Model 2: age, sex, BMI, smoking status, and drinking status were adjusted.

Model 3: Model 2 plus adjustment for PA, DM, CHD, and cancer.

Model 4: Model 3 plus adjustment for ALT, AST, Cr, TC, TG, HDL-C, LDL-C, ALP, TSH, FPG, serum potassium, serum calcium, serum phosphorus, serum sodium, 24-h urinary potassium, 24-h urinary calcium,24-h urinary phosphorus, 24-h urinary sodium, PTH, and 25-hydroxyvitamin D.

Model 5: Model 4 plus adjustment for use of statins, aspirin, diuretics, beta-blockers, calcium channel blockers, ACEIs/ARBs, oral hypoglycemic agents, and insulin.

Abbreviations: PAC, plasma aldosterone concentration; BMD, bone mineral density; Neck, neck of the femur; Wards, Ward's triangle; MOF, major osteoporotic fracture; HF, hip fracture; β, regression coefficient; OR, odds ratio; CI, confidence interval.

Other abbreviations, see Table 1

**Table S10.** Sensitivity analysis of the relationship between PAC with BMD, FRAX scores, and osteoporosis was performed after excluding patients with cancer.

| Exposure | Model 1 | Model 2 | Model 3 | Model 4 | Model 5 |
| --- | --- | --- | --- | --- | --- |
|  | β / OR (95% CI) | β / OR (95% CI) | β / OR (95% CI) | β / OR (95% CI) | β / OR (95% CI) |
| **Lumbar 1** |  |  |  |  |  |
| PAC (per 1-ng/dL increase) | -0.08 (-0.09, -0.07) | -0.08 (-0.09, -0.06) | -0.06 (-0.08, -0.05) | -0.06 (-0.08, -0.05) | -0.06 (-0.08, -0.05) |
| Tertiles of PAC |  |  |  |  |  |
| Tertile 1 | Reference | Reference | Reference | Reference | Reference |
| Tertile 2 | -0.35 (-0.55, -0.16) | -0.38 (-0.57, -0.19) | -0.37 (-0.56, -0.18) | -0.39 (-0.58, -0.20) | -0.39 (-0.58, -0.20) |
| Tertile 3 | -1.43 (-1.63, -1.24) | -1.33 (-1.53, -1.14) | -1.21 (-1.44, -0.98) | -1.21 (-1.44, -0.97) | -1.19 (-1.42, -0.95) |
| P for trend | <0.001 | <0.001 | <0.001 | <0.001 | <0.001 |
| **Lumbar 2** |  |  |  |  |  |
| PAC (per 1-ng/dL increase) | -0.08 (-0.09, -0.06) | -0.07 (-0.08, -0.06) | -0.06 (-0.07, -0.04) | -0.06 (-0.07, -0.04) | -0.06 (-0.07, -0.04) |
| Tertiles of PAC |  |  |  |  |  |
| Tertile 1 | Reference | Reference | Reference | Reference | Reference |
| Tertile 2 | -0.44 (-0.64, -0.24) | -0.48 (-0.67, -0.29) | -0.47 (-0.66, -0.27) | -0.48 (-0.68, -0.29) | -0.49 (-0.68, -0.30) |
| Tertile 3 | -1.45 (-1.65, -1.25) | -1.34 (-1.53, -1.15) | -1.21 (-1.44, -0.97) | -1.21 (-1.45, -0.98) | -1.21 (-1.44, -0.97) |
| P for trend | <0.001 | <0.001 | <0.001 | <0.001 | <0.001 |
| **Lumbar 3** |  |  |  |  |  |
| PAC (per 1-ng/dL increase) | -0.08 (-0.10, -0.07) | -0.08 (-0.09, -0.06) | -0.06 (-0.07, -0.04) | -0.06 (-0.08, -0.05) | -0.06 (-0.08, -0.05) |
| Tertiles of PAC |  |  |  |  |  |
| Tertile 1 | Reference | Reference | Reference | Reference | Reference |
| Tertile 2 | -0.47 (-0.67, -0.26) | -0.49 (-0.69, -0.30) | -0.47 (-0.67, -0.27) | -0.49 (-0.69, -0.29) | -0.50 (-0.70, -0.30) |
| Tertile 3 | -1.56 (-1.77, -1.35) | -1.43 (-1.63, -1.23) | -1.25 (-1.49, -1.01) | -1.26 (-1.51, -1.02) | -1.27 (-1.52, -1.03) |
| P for trend | <0.001 | <0.001 | <0.001 | <0.001 | <0.001 |
| **Lumbar 4** |  |  |  |  |  |
| PAC (per 1-ng/dL increase) | -0.10 (-0.11, -0.08) | -0.09 (-0.10, -0.08) | -0.07 (-0.09, -0.06) | -0.08 (-0.09, -0.06) | -0.08 (-0.09, -0.06) |
| Tertiles of PAC |  |  |  |  |  |
| Tertile 1 | Reference | Reference | Reference | Reference | Reference |
| Tertile 2 | -0.50 (-0.71, -0.29) | -0.52 (-0.72, -0.32) | -0.49 (-0.69, -0.29) | -0.51 (-0.72, -0.31) | -0.52 (-0.72, -0.31) |
| Tertile 3 | -1.63 (-1.84, -1.42) | -1.51 (-1.71, -1.30) | -1.31 (-1.55, -1.06) | -1.32 (-1.57, -1.07) | -1.31 (-1.56, -1.06) |
| P for trend | <0.001 | <0.001 | <0.001 | <0.001 | <0.001 |
| **Neck** |  |  |  |  |  |
| PAC (per 1-ng/dL increase) | -0.07 (-0.07, -0.06) | -0.07 (-0.07, -0.06) | -0.05 (-0.06, -0.04) | -0.05 (-0.06, -0.04) | -0.05 (-0.06, -0.04) |
| Tertiles of PAC |  |  |  |  |  |
| Tertile 1 | Reference | Reference | Reference | Reference | Reference |
| Tertile 2 | -0.41 (-0.54, -0.28) | -0.46 (-0.58, -0.34) | -0.43 (-0.55, -0.30) | -0.45 (-0.57, -0.32) | -0.44 (-0.57, -0.32) |
| Tertile 3 | -1.14 (-1.27, -1.01) | -1.13 (-1.25, -1.00) | -0.97 (-1.12, -0.82) | -0.95 (-1.10, -0.80) | -0.94 (-1.09, -0.79) |
| P for trend | <0.001 | <0.001 | <0.001 | <0.001 | <0.001 |
| **Wards** |  |  |  |  |  |
| PAC (per 1-ng/dL increase) | -0.07 (-0.08, -0.06) | -0.07 (-0.08, -0.07) | -0.06 (-0.07, -0.05) | -0.06 (-0.07, -0.05) | -0.06 (-0.07, -0.05) |
| Tertiles of PAC |  |  |  |  |  |
| Tertile 1 | Reference | Reference | Reference | Reference | Reference |
| Tertile 2 | -0.44 (-0.59, -0.30) | -0.50 (-0.64, -0.36) | -0.46 (-0.60, -0.32) | -0.49 (-0.63, -0.35) | -0.49 (-0.63, -0.35) |
| Tertile 3 | -1.28 (-1.43, -1.13) | -1.29 (-1.43, -1.15) | -1.09 (-1.26, -0.92) | -1.07 (-1.24, -0.90) | -1.06 (-1.23, -0.89) |
| P for trend | <0.001 | <0.001 | <0.001 | <0.001 | <0.001 |
| **Total** |  |  |  |  |  |
| PAC (per 1-ng/dL increase) | -0.08 (-0.09, -0.07) | -0.08 (-0.08, -0.07) | -0.06 (-0.07, -0.05) | -0.06 (-0.07, -0.05) | -0.06 (-0.07, -0.05) |
| Tertiles of PAC |  |  |  |  |  |
| Tertile 1 | Reference | Reference | Reference | Reference | Reference |
| Tertile 2 | -0.49 (-0.61, -0.37) | -0.53 (-0.64, -0.42) | -0.49 (-0.61, -0.38) | -0.50 (-0.62, -0.39) | -0.51 (-0.62, -0.40) |
| Tertile 3 | -1.33 (-1.45, -1.21) | -1.30 (-1.41, -1.18) | -1.12 (-1.26, -0.98) | -1.10 (-1.24, -0.97) | -1.11 (-1.25, -0.97) |
| P for trend | <0.001 | <0.001 | <0.001 | <0.001 | <0.001 |
| **MOF** |  |  |  |  |  |
| PAC (per 1-ng/dL increase) | 0.18 (0.15, 0.20) | 0.17 (0.15, 0.19) | 0.14 (0.11, 0.16) | 0.14 (0.11, 0.16) | 0.14 (0.11, 0.17) |
| Tertiles of PAC |  |  |  |  |  |
| Tertile 1 | Reference | Reference | Reference | Reference | Reference |
| Tertile 2 | 0.40 (0.05, 0.75) | 0.47 (0.15, 0.79) | 0.34 (0.01, 0.66) | 0.42 (0.09, 0.74) | 0.42 (0.10, 0.75) |
| Tertile 3 | 2.72 (2.37, 3.07) | 2.61 (2.29, 2.94) | 2.09 (1.69, 2.48) | 2.10 (1.70, 2.50) | 2.10 (1.70, 2.50) |
| P for trend | <0.001 | <0.001 | <0.001 | <0.001 | <0.001 |
| **HF** |  |  |  |  |  |
| PAC (per 1-ng/dL increase) | 0.14 (0.12, 0.15) | 0.13 (0.12, 0.15) | 0.11 (0.09, 0.13) | 0.11 (0.09, 0.13) | 0.11 (0.09, 0.13) |
| Tertiles of PAC |  |  |  |  |  |
| Tertile 1 | Reference | Reference | Reference | Reference | Reference |
| Tertile 2 | 0.25 (-0.03, 0.53) | 0.27 (0.01, 0.54) | 0.17 (-0.10, 0.45) | 0.24 (-0.03, 0.51) | 0.24 (-0.03, 0.51) |
| Tertile 3 | 1.96 (1.68, 2.24) | 1.96 (1.69, 2.23) | 1.55 (1.22, 1.88) | 1.57 (1.24, 1.90) | 1.57 (1.24, 1.91) |
| P for trend | <0.0001 | <0.001 | <0.001 | <0.001 | <0.001 |
| **Osteoporosis** |  |  |  |  |  |
| PAC (per 1-ng/dL increase) | 1.11 (1.09, 1.14) | 1.11 (1.09, 1.14) | 1.08 (1.05, 1.12) | 1.09 (1.06, 1.12) | 1.09 (1.06, 1.12) |
| Tertiles of PAC |  |  |  |  |  |
| Tertile 1 | Reference | Reference | Reference | Reference | Reference |
| Tertile 2 | 1.34 (0.93, 1.92) | 1.40 (0.96, 2.03) | 1.34 (0.92, 1.96) | 1.39 (0.95, 2.05) | 1.40 (0.95, 2.07) |
| Tertile 3 | 5.17 (3.72, 7.18) | 5.16 (3.67, 7.26) | 3.99 (2.66, 5.99) | 4.12 (2.72, 6.24) | 4.04 (2.65, 6.15) |
| P for trend | <0.001 | <0.001 | <0.001 | <0.001 | <0.001 |

Model 1: no covariates were adjusted.

Model 2: age, sex, BMI, smoking status, and drinking status were adjusted.

Model 3: Model 2 plus adjustment for PA, DM, and CHD.

Model 4: Model 3 plus adjustment for ALT, AST, Cr, TC, TG, HDL-C, LDL-C, ALP, TSH, FPG, serum potassium, serum calcium, serum phosphorus, serum sodium, 24-h urinary potassium, 24-h urinary calcium,24-h urinary phosphorus, 24-h urinary sodium, PTH, and 25-hydroxyvitamin D.

Model 5: Model 4 plus adjustment for use of statins, aspirin, diuretics, beta-blockers, calcium channel blockers, ACEIs/ARBs, oral hypoglycemic agents, and insulin.

Abbreviations: PAC, plasma aldosterone concentration; BMD, bone mineral density; Neck, neck of the femur; Wards, Ward's triangle; MOF, major osteoporotic fracture; HF, hip fracture; β, regression coefficient; OR, odds ratio; CI, confidence interval.

Other abbreviations, see Table 1.

**Table S11.** Sensitivity analysis of the relationship between PAC with BMD, FRAX scores, and osteoporosis was performed after excluding patients with 25-hydroxyvitamin D < 20 nmol/L.

| Exposure | Model 1 | Model 2 | Model 3 | Model 4 | Model 5 |
| --- | --- | --- | --- | --- | --- |
|  | β / OR (95% CI) | β / OR (95% CI) | β / OR (95% CI) | β / OR (95% CI) | β / OR (95% CI) |
| **Lumbar 1** |  |  |  |  |  |
| PAC (per 1-ng/dL increase) | -0.07 (-0.09, -0.05) | -0.07 (-0.08, -0.05) | -0.05 (-0.08, -0.03) | -0.06 (-0.08, -0.04) | -0.06 (-0.08, -0.04) |
| Tertiles of PAC |  |  |  |  |  |
| Tertile 1 | Reference | Reference | Reference | Reference | Reference |
| Tertile 2 | -0.35 (-0.63, -0.07) | -0.35 (-0.62, -0.08) | -0.31 (-0.59, -0.04) | -0.33 (-0.61, -0.05) | -0.36 (-0.63, -0.08) |
| Tertile 3 | -1.24 (-1.53, -0.95) | -1.15 (-1.43, -0.86) | -0.99 (-1.33, -0.65) | -0.97 (-1.32, -0.62) | -1.01 (-1.37, -0.66) |
| P for trend | <0.001 | <0.001 | <0.001 | <0.001 | <0.001 |
| **Lumbar 2** |  |  |  |  |  |
| PAC (per 1-ng/dL increase) | -0.06 (-0.08, -0.05) | -0.06 (-0.08, -0.04) | -0.05 (-0.07, -0.02) | -0.05 (-0.07, -0.03) | -0.05 (-0.07, -0.03) |
| Tertiles of PAC |  |  |  |  |  |
| Tertile 1 | Reference | Reference | Reference | Reference | Reference |
| Tertile 2 | -0.46 (-0.74, -0.18) | -0.47 (-0.74, -0.20) | -0.43 (-0.70, -0.15) | -0.45 (-0.73, -0.17) | -0.48 (-0.76, -0.21) |
| Tertile 3 | -1.28 (-1.57, -0.99) | -1.18 (-1.47, -0.89) | -1.00 (-1.34, -0.66) | -0.99 (-1.34, -0.64) | -1.03 (-1.38, -0.68) |
| P for trend | <0.001 | <0.001 | <0.001 | <0.001 | <0.001 |
| **Lumbar 3** |  |  |  |  |  |
| PAC (per 1-ng/dL increase) | -0.07 (-0.09, -0.05) | -0.07 (-0.08, -0.05) | -0.05 (-0.07, -0.03) | -0.05 (-0.07, -0.03) | -0.05 (-0.08, -0.03) |
| Tertiles of PAC |  |  |  |  |  |
| Tertile 1 | Reference | Reference | Reference | Reference | Reference |
| Tertile 2 | -0.53 (-0.82, -0.24) | -0.53 (-0.81, -0.26) | -0.49 (-0.77, -0.21) | -0.50 (-0.79, -0.22) | -0.54 (-0.83, -0.26) |
| Tertile 3 | -1.41 (-1.71, -1.11) | -1.30 (-1.59, -1.00) | -1.10 (-1.45, -0.75) | -1.12 (-1.47, -0.76) | -1.18 (-1.54, -0.81) |
| P for trend | <0.001 | <0.001 | <0.001 | <0.001 | <0.001 |
| **Lumbar 4** |  |  |  |  |  |
| PAC (per 1-ng/dL increase) | -0.09 (-0.11, -0.07) | -0.08 (-0.10, -0.07) | -0.07 (-0.09, -0.05) | -0.07 (-0.10, -0.05) | -0.07 (-0.10, -0.05) |
| Tertiles of PAC |  |  |  |  |  |
| Tertile 1 | Reference | Reference | Reference | Reference | Reference |
| Tertile 2 | -0.52 (-0.81, -0.23) | -0.53 (-0.81, -0.25) | -0.48 (-0.76, -0.20) | -0.51 (-0.80, -0.22) | -0.53 (-0.82, -0.25) |
| Tertile 3 | -1.54 (-1.85, -1.24) | -1.44 (-1.74, -1.14) | -1.22 (-1.58, -0.87) | -1.25 (-1.61, -0.89) | -1.30 (-1.66, -0.93) |
| P for trend | <0.001 | <0.001 | <0.001 | <0.001 | <0.001 |
| **Neck** |  |  |  |  |  |
| PAC (per 1-ng/dL increase) | -0.05 (-0.06, -0.04) | -0.06 (-0.07, -0.04) | -0.04 (-0.06, -0.03) | -0.04 (-0.06, -0.03) | -0.04 (-0.06, -0.03) |
| Tertiles of PAC |  |  |  |  |  |
| Tertile 1 | Reference | Reference | Reference | Reference | Reference |
| Tertile 2 | -0.41 (-0.58, -0.24) | -0.43 (-0.59, -0.27) | -0.38 (-0.54, -0.22) | -0.39 (-0.55, -0.23) | -0.39 (-0.55, -0.22) |
| Tertile 3 | -0.94 (-1.12, -0.76) | -0.97 (-1.14, -0.80) | -0.81 (-1.01, -0.61) | -0.80 (-1.00, -0.59) | -0.80 (-1.01, -0.59) |
| P for trend | <0.001 | <0.001 | <0.001 | <0.001 | <0.001 |
| **Wards** |  |  |  |  |  |
| PAC (per 1-ng/dL increase) | -0.06 (-0.07, -0.05) | -0.06 (-0.08, -0.05) | -0.05 (-0.07, -0.04) | -0.05 (-0.06, -0.04) | -0.05 (-0.06, -0.03) |
| Tertiles of PAC |  |  |  |  |  |
| Tertile 1 | Reference | Reference | Reference | Reference | Reference |
| Tertile 2 | -0.46 (-0.65, -0.26) | -0.50 (-0.68, -0.31) | -0.46 (-0.64, -0.27) | -0.50 (-0.69, -0.31) | -0.49 (-0.68, -0.31) |
| Tertile 3 | -1.11 (-1.32, -0.91) | -1.15 (-1.34, -0.95) | -1.00 (-1.23, -0.76) | -1.01 (-1.24, -0.77) | -1.00 (-1.25, -0.76) |
| P for trend | <0.001 | <0.001 | <0.001 | <0.001 | <0.001 |
| **Total** |  |  |  |  |  |
| PAC (per 1-ng/dL increase) | -0.06 (-0.07, -0.05) | -0.06 (-0.07, -0.05) | -0.05 (-0.06, -0.04) | -0.05 (-0.06, -0.04) | -0.05 (-0.06, -0.04) |
| Tertiles of PAC |  |  |  |  |  |
| Tertile 1 | Reference | Reference | Reference | Reference | Reference |
| Tertile 2 | -0.52 (-0.68, -0.36) | -0.54 (-0.69, -0.39) | -0.49 (-0.65, -0.34) | -0.50 (-0.65, -0.35) | -0.51 (-0.66, -0.36) |
| Tertile 3 | -1.16 (-1.33, -0.99) | -1.17 (-1.33, -1.01) | -1.00 (-1.19, -0.81) | -1.02 (-1.21, -0.83) | -1.03 (-1.23, -0.84) |
| P for trend | <0.001 | <0.001 | <0.001 | <0.001 | <0.001 |
| **MOF** |  |  |  |  |  |
| PAC (per 1-ng/dL increase) | 0.15 (0.12, 0.18) | 0.14 (0.12, 0.17) | 0.11 (0.08, 0.14) | 0.11 (0.08, 0.14) | 0.11 (0.08, 0.14) |
| Tertiles of PAC |  |  |  |  |  |
| Tertile 1 | Reference | Reference | Reference | Reference | Reference |
| Tertile 2 | 0.37 (-0.06, 0.81) | 0.37 (-0.04, 0.77) | 0.22 (-0.19, 0.64) | 0.27 (-0.15, 0.69) | 0.29 (-0.12, 0.71) |
| Tertile 3 | 2.52 (2.07, 2.98) | 2.39 (1.96, 2.83) | 1.88 (1.37, 2.40) | 1.94 (1.42, 2.47) | 2.00 (1.46, 2.53) |
| P for trend | <0.001 | <0.001 | <0.001 | <0.001 | <0.001 |
| **HF** |  |  |  |  |  |
| PAC (per 1-ng/dL increase) | 0.11 (0.09, 0.14) | 0.11 (0.09, 0.13) | 0.09 (0.06, 0.11) | 0.09 (0.06, 0.11) | 0.09 (0.06, 0.12) |
| Tertiles of PAC |  |  |  |  |  |
| Tertile 1 | Reference | Reference | Reference | Reference | Reference |
| Tertile 2 | 0.23 (-0.11, 0.58) | 0.20 (-0.13, 0.54) | 0.10 (-0.25, 0.44) | 0.12 (-0.23, 0.47) | 0.14 (-0.21, 0.49) |
| Tertile 3 | 1.85 (1.48, 2.21) | 1.82 (1.46, 2.18) | 1.44 (1.01, 1.87) | 1.50 (1.06, 1.93) | 1.55 (1.10, 2.00) |
| P for trend | <0.001 | <0.001 | <0.001 | <0.001 | <0.001 |
| **Osteoporosis** |  |  |  |  |  |
| PAC (per 1-ng/dL increase) | 1.11 (1.07, 1.14) | 1.11 (1.07, 1.15) | 1.09 (1.05, 1.13) | 1.09 (1.04, 1.13) | 1.09 (1.05, 1.14) |
| Tertiles of PAC |  |  |  |  |  |
| Tertile 1 | Reference | Reference | Reference | Reference | Reference |
| Tertile 2 | 1.65 (0.99, 2.75) | 1.66 (0.98, 2.80) | 1.57 (0.92, 2.69) | 1.51 (0.86, 2.63) | 1.71 (0.96, 3.04) |
| Tertile 3 | 4.98 (3.08, 8.04) | 5.01 (3.02, 8.30) | 3.90 (2.15, 7.10) | 4.04 (2.17, 7.53) | 4.91 (2.53, 9.52) |
| P for trend | <0.001 | <0.001 | <0.001 | <0.001 | <0.001 |

Model 1: no covariates were adjusted.

Model 2: age, sex, BMI, smoking status, and drinking status were adjusted.

Model 3: Model 2 plus adjustment for PA, DM, CHD, and cancer.

Model 4: Model 3 plus adjustment for ALT, AST, Cr, TC, TG, HDL-C, LDL-C, ALP, TSH, FPG, serum potassium, serum calcium, serum phosphorus, serum sodium, 24-h urinary potassium, 24-h urinary calcium,24-h urinary phosphorus, 24-h urinary sodium, PTH, and 25-hydroxyvitamin D.

Model 5: Model 4 plus adjustment for use of statins, aspirin, diuretics, beta-blockers, calcium channel blockers, ACEIs/ARBs, oral hypoglycemic agents, and insulin.

Abbreviations: PAC, plasma aldosterone concentration; BMD, bone mineral density; Neck, neck of the femur; Wards, Ward's triangle; MOF, major osteoporotic fracture; HF, hip fracture; β, regression coefficient; OR, odds ratio; CI, confidence interval.

Other abbreviations, see Table 1.

**Table S12.** Sensitivity analysis of the relationship between PAC with BMD, FRAX scores, and osteoporosis was performed after excluding patients with BMI > 30 kg/m^2^.

| Exposure | Model 1 | Model 2 | Model 3 | Model 4 | Model 5 |
| --- | --- | --- | --- | --- | --- |
|  | β / OR (95% CI) | β / OR (95% CI) | β / OR (95% CI) | β / OR (95% CI) | β / OR (95% CI) |
| **Lumbar 1** |  |  |  |  |  |
| PAC (per 1-ng/dL increase) | -0.07 (-0.08, -0.05) | -0.07 (-0.08, -0.05) | -0.05 (-0.07, -0.04) | -0.05 (-0.07, -0.04) | -0.05 (-0.07, -0.04) |
| Tertiles of PAC |  |  |  |  |  |
| Tertile 1 | Reference | Reference | Reference | Reference | Reference |
| Tertile 2 | -0.42 (-0.62, -0.21) | -0.45 (-0.65, -0.25) | -0.44 (-0.64, -0.23) | -0.45 (-0.65, -0.24) | -0.46 (-0.66, -0.25) |
| Tertile 3 | -1.37 (-1.58, -1.16) | -1.30 (-1.50, -1.10) | -1.16 (-1.40, -0.91) | -1.16 (-1.41, -0.91) | -1.16 (-1.40, -0.91) |
| P for trend | <0.001 | <0.001 | <0.001 | <0.001 | <0.001 |
| **Lumbar 2** |  |  |  |  |  |
| PAC (per 1-ng/dL increase) | -0.06 (-0.08, -0.05) | -0.06 (-0.07, -0.05) | -0.05 (-0.06, -0.03) | -0.05 (-0.06, -0.03) | -0.05 (-0.06, -0.04) |
| Tertiles of PAC |  |  |  |  |  |
| Tertile 1 | Reference | Reference | Reference | Reference | Reference |
| Tertile 2 | -0.50 (-0.71, -0.29) | -0.54 (-0.74, -0.34) | -0.52 (-0.73, -0.32) | -0.53 (-0.73, -0.33) | -0.54 (-0.75, -0.34) |
| Tertile 3 | -1.38 (-1.58, -1.17) | -1.30 (-1.50, -1.10) | -1.14 (-1.38, -0.89) | -1.15 (-1.39, -0.90) | -1.16 (-1.40, -0.91) |
| P for trend | <0.001 | <0.001 | <0.001 | <0.001 | <0.001 |
| **Lumbar 3** |  |  |  |  |  |
| PAC (per 1-ng/dL increase) | -0.07 (-0.08, -0.05) | -0.06 (-0.08, -0.05) | -0.04 (-0.06, -0.03) | -0.05 (-0.06, -0.03) | -0.05 (-0.06, -0.03) |
| Tertiles of PAC |  |  |  |  |  |
| Tertile 1 | Reference | Reference | Reference | Reference | Reference |
| Tertile 2 | -0.50 (-0.72, -0.28) | -0.54 (-0.75, -0.33) | -0.50 (-0.71, -0.29) | -0.51 (-0.72, -0.30) | -0.52 (-0.73, -0.31) |
| Tertile 3 | -1.46 (-1.67, -1.24) | -1.35 (-1.56, -1.14) | -1.13 (-1.38, -0.88) | -1.14 (-1.39, -0.88) | -1.16 (-1.42, -0.90) |
| P for trend | <0.001 | <0.001 | <0.001 | <0.001 | <0.001 |
| **Lumbar 4** |  |  |  |  |  |
| PAC (per 1-ng/dL increase) | -0.08 (-0.09, -0.07) | -0.08 (-0.09, -0.06) | -0.06 (-0.07, -0.04) | -0.06 (-0.08, -0.04) | -0.06 (-0.08, -0.05) |
| Tertiles of PAC |  |  |  |  |  |
| Tertile 1 | Reference | Reference | Reference | Reference | Reference |
| Tertile 2 | -0.51 (-0.74, -0.29) | -0.55 (-0.77, -0.33) | -0.51 (-0.73, -0.29) | -0.52 (-0.74, -0.30) | -0.53 (-0.75, -0.31) |
| Tertile 3 | -1.55 (-1.77, -1.33) | -1.45 (-1.67, -1.24) | -1.21 (-1.48, -0.95) | -1.21 (-1.48, -0.95) | -1.23 (-1.50, -0.96) |
| P for trend | <0.001 | <0.001 | <0.001 | <0.001 | <0.001 |
| **Neck** |  |  |  |  |  |
| PAC (per 1-ng/dL increase) | -0.05 (-0.06, -0.05) | -0.05 (-0.06, -0.05) | -0.04 (-0.05, -0.03) | -0.04 (-0.05, -0.03) 1 | -0.04 (-0.05, -0.03) |
| Tertiles of PAC |  |  |  |  |  |
| Tertile 1 | Reference | Reference | Reference | Reference | Reference |
| Tertile 2 | -0.38 (-0.51, -0.24) | -0.43 (-0.56, -0.30) | -0.40 (-0.53, -0.27) | -0.41 (-0.54, -0.27) | -0.41 (-0.54, -0.28) |
| Tertile 3 | -1.05 (-1.19, -0.92) | -1.06 (-1.19, -0.93) | -0.90 (-1.05, -0.74) | -0.87 (-1.03, -0.71) | -0.86 (-1.02, -0.71) |
| P for trend | <0.001 | <0.001 | <0.001 | <0.001 | <0.001 |
| **Wards** |  |  |  |  |  |
| PAC (per 1-ng/dL increase) | -0.06 (-0.07, -0.05) | -0.06 (-0.07, -0.05) | -0.05 (-0.06, -0.04) | -0.04 (-0.06, -0.03) | -0.04 (-0.06, -0.03) |
| Tertiles of PAC |  |  |  |  |  |
| Tertile 1 | Reference | Reference | Reference | Reference | Reference |
| Tertile 2 | -0.35 (-0.51, -0.19) | -0.42 (-0.57, -0.27) | -0.38 (-0.53, -0.23) | -0.39 (-0.54, -0.25) | -0.40 (-0.55, -0.25) |
| Tertile 3 | -1.17 (-1.32, -1.01) | -1.19 (-1.34, -1.04) | -1.00 (-1.18, -0.82) | -0.97 (-1.15, -0.79) | -0.98 (-1.16, -0.80) |
| P for trend | <0.001 | <0.001 | <0.001 | <0.001 | <0.001 |
| **Total** |  |  |  |  |  |
| PAC (per 1-ng/dL increase) | -0.06 (-0.07, -0.06) | -0.07 (-0.07, -0.06) | -0.05 (-0.06, -0.04) | -0.05 (-0.06, -0.04) | -0.05 (-0.06, -0.04) |
| Tertiles of PAC |  |  |  |  |  |
| Tertile 1 | Reference | Reference | Reference | Reference | Reference |
| Tertile 2 | -0.44 (-0.57, -0.31) | -0.49 (-0.61, -0.37) | -0.45 (-0.58, -0.33) | -0.45 (-0.57, -0.32) | -0.45 (-0.57, -0.33) |
| Tertile 3 | -1.25 (-1.38, -1.12) | -1.24 (-1.37, -1.12) | -1.05 (-1.20, -0.90) | -1.02 (-1.17, -0.88) | -1.04 (-1.19, -0.89) |
| P for trend | <0.001 | <0.001 | <0.001 | <0.001 | <0.001 |
| **MOF** |  |  |  |  |  |
| PAC (per 1-ng/dL increase) | 0.16 (0.14, 0.18) | 0.16 (0.14, 0.18) | 0.12 (0.09, 0.15) | 0.12 (0.10, 0.15) | 0.13 (0.10, 0.15) |
| Tertiles of PAC |  |  |  |  |  |
| Tertile 1 | Reference | Reference | Reference | Reference | Reference |
| Tertile 2 | 0.40 (-0.01, 0.81) | 0.49 (0.10, 0.87) | 0.35 (-0.04, 0.74) | 0.41 (0.03, 0.80) | 0.42 (0.03, 0.81) |
| Tertile 3 | 2.75 (2.34, 3.15) | 2.66 (2.28, 3.05) | 2.03 (1.56, 2.49) | 2.06 (1.60, 2.53) | 2.09 (1.62, 2.55) |
| P for trend | <0.001 | <0.001 | <0.001 | <0.001 | <0.001 |
| **HF** |  |  |  |  |  |
| PAC (per 1-ng/dL increase) | 0.13 (0.11, 0.15) | 0.13 (0.11, 0.15) | 0.10 (0.08, 0.12) | 0.11 (0.08, 0.13) | 0.11 (0.08, 0.13) |
| Tertiles of PAC |  |  |  |  |  |
| Tertile 1 | Reference | Reference | Reference | Reference | Reference |
| Tertile 2 | 0.27 (-0.06, 0.61) | 0.31 (-0.02, 0.64) | 0.21 (-0.12, 0.54) | 0.26 (-0.07, 0.59) | 0.27 (-0.06, 0.60) |
| Tertile 3 | 2.01 (1.67, 2.34) | 2.05 (1.73, 2.38) | 1.58 (1.19, 1.97) | 1.62 (1.22, 2.01) | 1.64 (1.24, 2.03) |
| P for trend | <0.001 | <0.001 | <0.001 | <0.001 | <0.001 |
| **Osteoporosis** |  |  |  |  |  |
| PAC (per 1-ng/dL increase) | 1.09 (1.07, 1.12) | 1.09 (1.07, 1.12) | 1.07 (1.04, 1.09) | 1.07 (1.04, 1.10) | 1.07 (1.04, 1.10) |
| Tertiles of PAC |  |  |  |  |  |
| Tertile 1 | Reference | Reference | Reference | Reference | Reference |
| Tertile 2 | 1.42 (0.96, 2.08) | 1.50 (1.01, 2.23) | 1.43 (0.96, 2.13) | 1.45 (0.97, 2.18) | 1.49 (0.99, 2.26) |
| Tertile 3 | 4.99 (3.52, 7.07) | 5.05 (3.51, 7.26) | 3.84 (2.50, 5.89) | 3.93 (2.53, 6.11) | 4.08 (2.60, 6.40) |
| P for trend | <0.001 | <0.001 | <0.001 | <0.001 | <0.001 |

Model 1: no covariates were adjusted.

Model 2: age, sex, BMI, smoking status, and drinking status were adjusted.

Model 3: Model 2 plus adjustment for PA, DM, CHD, and cancer.

Model 4: Model 3 plus adjustment for ALT, AST, Cr, TC, TG, HDL-C, LDL-C, ALP, TSH, FPG, serum potassium, serum calcium, serum phosphorus, serum sodium, 24-h urinary potassium, 24-h urinary calcium,24-h urinary phosphorus, 24-h urinary sodium, PTH, and 25-hydroxyvitamin D.

Model 5: Model 4 plus adjustment for use of statins, aspirin, diuretics, beta-blockers, calcium channel blockers, ACEIs/ARBs, oral hypoglycemic agents, and insulin.

Abbreviations: PAC, plasma aldosterone concentration; BMD, bone mineral density; Neck, neck of the femur; Wards, Ward's triangle; MOF, major osteoporotic fracture; HF, hip fracture; β, regression coefficient; OR, odds ratio; CI, confidence interval.

Other abbreviations, see Table 1.

**Table S13.** Sensitivity analysis of the relationship between PAC with BMD, FRAX scores, and osteoporosis was performed after excluding patients with age > 75 years.

| Exposure | Model 1 | Model 2 | Model 3 | Model 4 | Model 5 |
| --- | --- | --- | --- | --- | --- |
|  | β / OR (95% CI) | β / OR (95% CI) | β / OR (95% CI) | β / OR (95% CI) | β / OR (95% CI) |
| **Lumbar 1** |  |  |  |  |  |
| PAC (per 1-ng/dL increase) | -0.07 (-0.08, -0.06) | -0.07 (-0.08, -0.06) | -0.05 (-0.07, -0.04) | -0.05 (-0.07, -0.04) | -0.05 (-0.07, -0.04) |
| Tertiles of PAC |  |  |  |  |  |
| Tertile 1 | Reference | Reference | Reference | Reference | Reference |
| Tertile 2 | -0.34 (-0.54, -0.14) | -0.37 (-0.56, -0.18) | -0.35 (-0.54, -0.16) | -0.38 (-0.57, -0.19) | -0.38 (-0.57, -0.19) |
| Tertile 3 | -1.35 (-1.55, -1.15) | -1.28 (-1.48, -1.09) | -1.12 (-1.35, -0.90) | -1.14 (-1.37, -0.91) | -1.13 (-1.36, -0.89) |
| P for trend | <0.001 | <0.001 | <0.001 | <0.001 | <0.001 |
| **Lumbar 2** |  |  |  |  |  |
| PAC (per 1-ng/dL increase) | -0.07 (-0.08, -0.05) | -0.06 (-0.08, -0.05) | -0.05 (-0.06, -0.03) | -0.05 (-0.06, -0.04) | -0.05 (-0.06, -0.04) |
| Tertiles of PAC |  |  |  |  |  |
| Tertile 1 | Reference | Reference | Reference | Reference | Reference |
| Tertile 2 | -0.41 (-0.61, -0.22) | -0.45 (-0.64, -0.26) | -0.43 (-0.62, -0.24) | -0.46 (-0.65, -0.27) | -0.47 (-0.66, -0.27) |
| Tertile 3 | -1.34 (-1.54, -1.14) | -1.27 (-1.46, -1.08) | -1.11 (-1.34, -0.88) | -1.13 (-1.36, -0.90) | -1.13 (-1.36, -0.90) |
| P for trend | <0.001 | <0.001 | <0.001 | <0.001 | <0.001 |
| **Lumbar 3** |  |  |  |  |  |
| PAC (per 1-ng/dL increase) | -0.07 (-0.08, -0.06) | -0.07 (-0.08, -0.05) | -0.05 (-0.06, -0.03) | -0.05 (-0.07, -0.04) | -0.05 (-0.07, -0.04) |
| Tertiles of PAC |  |  |  |  |  |
| Tertile 1 | Reference | Reference | Reference | Reference | Reference |
| Tertile 2 | -0.43 (-0.64, -0.22) | -0.47 (-0.66, -0.27) | -0.43 (-0.63, -0.23) | -0.47 (-0.67, -0.27) | -0.47 (-0.67, -0.28) |
| Tertile 3 | -1.45 (-1.66, -1.24) | -1.36 (-1.56, -1.16) | -1.16 (-1.40, -0.92) | -1.18 (-1.42, -0.94) | -1.19 (-1.43, -0.95) |
| P for trend | <0.001 | <0.001 | <0.001 | <0.001 | <0.001 |
| **Lumbar 4** |  |  |  |  |  |
| PAC (per 1-ng/dL increase) | -0.08 (-0.09, -0.07) | -0.08 (-0.09, -0.06) | -0.06 (-0.07, -0.04) | -0.06 (-0.08, -0.05) | -0.06 (-0.08, -0.05) |
| Tertiles of PAC |  |  |  |  |  |
| Tertile 1 | Reference | Reference | Reference | Reference | Reference |
| Tertile 2 | -0.45 (-0.66, -0.24) | -0.48 (-0.68, -0.28) | -0.44 (-0.64, -0.23) | -0.47 (-0.68, -0.27) | -0.47 (-0.68, -0.27) |
| Tertile 3 | -1.51 (-1.72, -1.30) | -1.43 (-1.64, -1.23) | -1.21 (-1.46, -0.97) | -1.23 (-1.47, -0.98) | -1.22 (-1.47, -0.97) |
| P for trend | <0.001 | <0.001 | <0.001 | <0.001 | <0.001 |
| **Neck** |  |  |  |  |  |
| PAC (per 1-ng/dL increase) | -0.06 (-0.07, -0.05) | -0.06 (-0.07, -0.05) | -0.04 (-0.05, -0.03) | -0.04 (-0.05, -0.03) | -0.04 (-0.05, -0.03) |
| Tertiles of PAC |  |  |  |  |  |
| Tertile 1 | Reference | Reference | Reference | Reference | Reference |
| Tertile 2 | -0.39 (-0.52, -0.26) | -0.43 (-0.56, -0.31) | -0.39 (-0.52, -0.27) | -0.41 (-0.54, -0.29) | -0.41 (-0.54, -0.29) |
| Tertile 3 | -1.08 (-1.21, -0.95) | -1.08 (-1.20, -0.95) | -0.90 (-1.05, -0.75) | -0.89 (-1.04, -0.74) | -0.88 (-1.03, -0.73) |
| P for trend | <0.001 | <0.001 | <0.001 | <0.001 | <0.001 |
| **Wards** |  |  |  |  |  |
| PAC (per 1-ng/dL increase) | -0.07 (-0.07, -0.06) | -0.07 (-0.08, -0.06) | -0.05 (-0.06, -0.04) | -0.05 (-0.06, -0.04) | -0.05 (-0.06, -0.04) |
| Tertiles of PAC |  |  |  |  |  |
| Tertile 1 | Reference | Reference | Reference | Reference | Reference |
| Tertile 2 | -0.41 (-0.56, -0.27) | -0.47 (-0.61, -0.33) | -0.42 (-0.56, -0.28) | -0.45 (-0.59, -0.31) | -0.45 (-0.59, -0.31) |
| Tertile 3 | -1.21 (-1.36, -1.06) | -1.24 (-1.38, -1.10) | -1.03 (-1.20, -0.86) | -1.02 (-1.19, -0.85) | -1.02 (-1.19, -0.85) |
| P for trend | <0.001 | <0.001 | <0.001 | <0.001 | <0.001 |
| **Total** |  |  |  |  |  |
| PAC (per 1-ng/dL increase) | -0.07 (-0.08, -0.06) | -0.07 (-0.08, -0.06) | -0.05 (-0.06, -0.04) | -0.05 (-0.06, -0.04) | -0.05 (-0.06, -0.04) |
| Tertiles of PAC |  |  |  |  |  |
| Tertile 1 | Reference | Reference | Reference | Reference | Reference |
| Tertile 2 | -0.49 (-0.61, -0.36) | -0.52 (-0.64, -0.41) | -0.47 (-0.59, -0.36) | -0.49 (-0.60, -0.37) | -0.49 (-0.61, -0.38) |
| Tertile 3 | -1.28 (-1.40, -1.16) | -1.26 (-1.38, -1.14) | -1.05 (-1.19, -0.91) | -1.05 (-1.18, -0.91) | -1.05 (-1.19, -0.92) |
| P for trend | <0.001 | <0.001 | <0.001 | <0.001 | <0.001 |
| **MOF** |  |  |  |  |  |
| PAC (per 1-ng/dL increase) | 0.16 (0.13, 0.18) | 0.16 (0.14, 0.18) | 0.12 (0.10, 0.14) | 0.13 (0.10, 0.15) | 0.13 (0.10, 0.15) |
| Tertiles of PAC |  |  |  |  |  |
| Tertile 1 | Reference | Reference | Reference | Reference | Reference |
| Tertile 2 | 0.31 (-0.04, 0.65) | 0.37 (0.04, 0.69) | 0.23 (-0.10, 0.56) | 0.31 (-0.02, 0.64) | 0.32 (-0.01, 0.64) |
| Tertile 3 | 2.47 (2.12, 2.82) | 2.45 (2.12, 2.78) | 1.91 (1.51, 2.30) | 1.95 (1.56, 2.34) | 1.96 (1.56, 2.36) |
| P for trend | <0.001 | <0.001 | <0.001 | <0.001 | <0.001 |
| **HF** |  |  |  |  |  |
| PAC (per 1-ng/dL increase) | 0.12 (0.11, 0.14) | 0.12 (0.11, 0.14) | 0.10 (0.08, 0.12) | 0.10 (0.08, 0.13) | 0.10 (0.08, 0.13) |
| Tertiles of PAC |  |  |  |  |  |
| Tertile 1 | Reference | Reference | Reference | Reference | Reference |
| Tertile 2 | 0.20 (-0.09, 0.48) | 0.21 (-0.07, 0.49) | 0.10 (-0.19, 0.38) | 0.16 (-0.12, 0.44) | 0.17 (-0.11, 0.45) |
| Tertile 3 | 1.84 (1.55, 2.13) | 1.88 (1.59, 2.16) | 1.44 (1.10, 1.78) | 1.48 (1.14, 1.81) | 1.49 (1.15, 1.83) |
| P for trend | <0.001 | <0.001 | <0.001 | <0.001 | <0.001 |
| **Osteoporosis** |  |  |  |  |  |
| PAC (per 1-ng/dL increase) | 1.09 (1.07, 1.11) | 1.09 (1.07, 1.12) | 1.06 (1.04, 1.09) | 1.07 (1.04, 1.09) | 1.07 (1.04, 1.10) |
| Tertiles of PAC |  |  |  |  |  |
| Tertile 1 | Reference | Reference | Reference | Reference | Reference |
| Tertile 2 | 1.19 (0.83, 1.71) | 1.24 (0.86, 1.80) | 1.19 (0.82, 1.72) | 1.24 (0.85, 1.81) | 1.25 (0.85, 1.84) |
| Tertile 3 | 4.30 (3.11, 5.94) | 4.38 (3.13, 6.12) | 3.34 (2.25, 4.95) | 3.42 (2.28, 5.11) | 3.45 (2.29, 5.19) |
| P for trend | <0.001 | <0.001 | <0.001 | <0.001 | <0.001 |

Model 1: no covariates were adjusted.

Model 2: age, sex, BMI, smoking status, and drinking status were adjusted.

Model 3: Model 2 plus adjustment for PA, DM, CHD, and cancer.

Model 4: Model 3 plus adjustment for ALT, AST, Cr, TC, TG, HDL-C, LDL-C, ALP, TSH, FPG, serum potassium, serum calcium, serum phosphorus, serum sodium, 24-h urinary potassium, 24-h urinary calcium,24-h urinary phosphorus, 24-h urinary sodium, PTH, and 25-hydroxyvitamin D.

Model 5: Model 4 plus adjustment for use of statins, aspirin, diuretics, beta-blockers, calcium channel blockers, ACEIs/ARBs, oral hypoglycemic agents, and insulin.

Abbreviations: PAC, plasma aldosterone concentration; BMD, bone mineral density; Neck, neck of the femur; Wards, Ward's triangle; MOF, major osteoporotic fracture; HF, hip fracture; β, regression coefficient; OR, odds ratio; CI, confidence interval.

Other abbreviations, see Table 1.

**Table S14.** Sensitivity analysis of the relationship between PAC with BMD, FRAX scores, and osteoporosis was performed after excluding patients with PA.

| Exposure | Model 1 | Model 2 | Model 3 | Model 4 | Model 5 |
| --- | --- | --- | --- | --- | --- |
|  | β / OR (95% CI) | β / OR (95% CI) | β / OR (95% CI) | β / OR (95% CI) | β / OR (95% CI) |
| **Lumbar 1** |  |  |  |  |  |
| PAC (per 1-ng/dL increase) | -0.07 (-0.08, -0.05) | -0.06 (-0.08, -0.05) | -0.07 (-0.08, -0.05) | -0.05 (-0.06, -0.03) | -0.05 (-0.06, -0.03) |
| Tertiles of PAC |  |  |  |  |  |
| Tertile 1 | Reference | Reference | Reference | Reference | Reference |
| Tertile 2 | -0.35 (-0.56, -0.13) | -0.38 (-0.59, -0.17) | -0.40 (-0.61, -0.19) | -0.40 (-0.61, -0.20) | -0.39 (-0.60, -0.18) |
| Tertile 3 | -1.33 (-1.54, -1.12) | -1.28 (-1.49, -1.07) | -1.30 (-1.50, -1.10) | -1.12 (-1.38, -0.87) | -1.11 (-1.35, -0.84) |
| P for trend | <0.001 | <0.001 | <0.001 | <0.001 | <0.001 |
| **Lumbar 2** |  |  |  |  |  |
| PAC (per 1-ng/dL increase) | -0.06 (-0.08, -0.05) | -0.06 (-0.07, -0.04) | -0.06 (-0.07, -0.05) | -0.04 (-0.06, -0.03) | -0.04 (-0.06, -0.03) |
| Tertiles of PAC |  |  |  |  |  |
| Tertile 1 | Reference | Reference | Reference | Reference | Reference |
| Tertile 2 | -0.41 (-0.63, -0.20) | -0.45 (-0.65, -0.24) | -0.47 (-0.67, -0.26) | -0.48 (-0.69, -0.27) | -0.48 (-0.69, -0.27) |
| Tertile 3 | -1.33 (-1.54, -1.12) | -1.27 (-1.48, -1.06) | -1.28 (-1.49, -1.07) | -1.13 (-1.39, -0.88) | -1.12 (-1.38, -0.86) |
| P for trend | <0.001 | <0.001 | <0.001 | <0.001 | <0.001 |
| **Lumbar 3** |  |  |  |  |  |
| PAC (per 1-ng/dL increase) | -0.07 (-0.08, -0.05) | -0.06 (-0.08, -0.05) | -0.07 (-0.08, -0.05) | -0.05 (-0.06, -0.03) | -0.04 (-0.06, -0.03) |
| Tertiles of PAC |  |  |  |  |  |
| Tertile 1 | Reference | Reference | Reference | Reference | Reference |
| Tertile 2 | -0.45 (-0.68, -0.23) | -0.49 (-0.70, -0.27) | -0.51 (-0.72, -0.29) | -0.50 (-0.72, -0.29) | -0.51 (-0.72, -0.29) |
| Tertile 3 | -1.47 (-1.70, -1.25) | -1.38 (-1.60, -1.17) | -1.41 (-1.62, -1.19) | -1.20 (-1.46, -0.93) | -1.20 (-1.46, -0.93) |
| P for trend | <0.001 | <0.001 | <0.001 | <0.001 | <0.001 |
| **Lumbar 4** |  |  |  |  |  |
| PAC (per 1-ng/dL increase) | -0.08 (-0.10, -0.07) | -0.08 (-0.09, -0.07) | -0.08 (-0.10, -0.07) 1 | -0.06 (-0.08, -0.05) | -0.06 (-0.08, -0.05) |
| Tertiles of PAC |  |  |  |  |  |
| Tertile 1 | Reference | Reference | Reference | Reference | Reference |
| Tertile 2 | -0.46 (-0.69, -0.23) | -0.48 (-0.71, -0.26) | -0.51 (-0.72, -0.28) | -0.50 (-0.72, -0.28) | -0.49 (-0.71, -0.26) |
| Tertile 3 | -1.54 (-1.76, -1.31) | -1.45 (-1.67, -1.23) | -1.47 (-1.69, -1.25) | -1.22 (-1.50, -0.96) | -1.21 (-1.48, -0.94) |
| P for trend | <0.001 | <0.001 | <0.001 | <0.001 | <0.001 |
| **Neck** |  |  |  |  |  |
| PAC (per 1-ng/dL increase) | -0.06 (-0.07, -0.05) | -0.06 (-0.07, -0.05) | -0.06 (-0.07, -0.05) | -0.04 (-0.05, -0.03) | -0.04 (-0.05, -0.03) |
| Tertiles of PAC |  |  |  |  |  |
| Tertile 1 | Reference | Reference | Reference | Reference | Reference |
| Tertile 2 | -0.39 (-0.53, -0.24) | -0.44 (-0.57, -0.30) | -0.45 (-0.58, -0.31) | -0.44 (-0.57, -0.30) | -0.43 (-0.57, -0.30) |
| Tertile 3 | -1.08 (-1.22, -0.93) | -1.09 (-1.23, -0.96) | -1.11 (-1.24, -0.97) | -0.89 (-1.05, -0.72) | -0.87 (-1.03, -0.70) |
| P for trend | <0.001 | <0.001 | <0.001 | <0.001 | <0.001 |
| **Wards** |  |  |  |  |  |
| PAC (per 1-ng/dL increase) | -0.07 (-0.07, -0.06) | -0.07 (-0.08, -0.06) | -0.07 (-0.08, -0.06) | -0.05 (-0.06, -0.04) | -0.05 (-0.06, -0.04) |
| Tertiles of PAC |  |  |  |  |  |
| Tertile 1 | Reference | Reference | Reference | Reference | Reference |
| Tertile 2 | -0.43 (-0.60, -0.27) | -0.48 (-0.64, -0.33) | -0.50 (-0.65, -0.35) | -0.51 (-0.66, -0.35) | -0.50 (-0.65, -0.34) |
| Tertile 3 | -1.23 (-1.39, -1.06) | -1.26 (-1.42, -1.10) | -1.27 (-1.43, -1.12) | -1.06 (-1.25, -0.87) | -1.04 (-1.23, -0.85) |
| P for trend | <0.001 | <0.001 | <0.001 | <0.001 | <0.001 |
| **Total** |  |  |  |  |  |
| PAC (per 1-ng/dL increase) | -0.07 (-0.08, -0.06) | -0.07 (-0.08, -0.06) | -0.07 (-0.08, -0.06) | -0.05 (-0.06, -0.04) | -0.05 (-0.06, -0.04) |
| Tertiles of PAC |  |  |  |  |  |
| Tertile 1 | Reference | Reference | Reference | Reference | Reference |
| Tertile 2 | -0.43 (-0.57, -0.29) | -0.47 (-0.60, -0.35) | -0.48 (-0.61, -0.35) | -0.46 (-0.58, -0.33) | -0.46 (-0.58, -0.33) |
| Tertile 3 | -1.28 (-1.42, -1.14) | -1.27 (-1.40, -1.15) | -1.28 (-1.41, -1.15) | -1.03 (-1.18, -0.88) | -1.03 (-1.19, -0.88) |
| P for trend | <0.001 | <0.001 | <0.001 | <0.001 | <0.001 |
| **MOF** |  |  |  |  |  |
| PAC (per 1-ng/dL increase) | 0.16 (0.14, 0.19) | 0.16 (0.14, 0.19) | 0.16 (0.14, 0.19) | 0.12 (0.09, 0.15) | 0.12 (0.09, 0.15) |
| Tertiles of PAC |  |  |  |  |  |
| Tertile 1 | Reference | Reference | Reference | Reference | Reference |
| Tertile 2 | 0.32 (-0.07, 0.71) | 0.39 (0.02, 0.75) | 0.39 (0.02, 0.75) | 0.38 (0.017, 0.75) | 0.36 (-0.01, 0.73) |
| Tertile 3 | 2.73 (2.33, 3.12) | 2.69 (2.32, 3.06) | 2.69 (2.32, 3.06) | 2.12 (1.68, 2.57) | 2.10 (1.65, 2.55) |
| P for trend | <0.001 | <0.001 | <0.001 | <0.001 | <0.001 |
| **HF** |  |  |  |  |  |
| PAC (per 1-ng/dL increase) | 0.12 (0.11, 0.14) | 0.12 (0.11, 0.14) | 0.10 (0.08, 0.12) | 0.10 (0.08, 0.13) | 0.10 (0.08, 0.13) |
| Tertiles of PAC |  |  |  |  |  |
| Tertile 1 | Reference | Reference | Reference | Reference | Reference |
| Tertile 2 | 0.19 (-013, 0.51) | 0.20 (-0.10, 0.51) | 0.20 (-0.10, 0.51) | 0.20 (-0.10, 0.52) | 0.19 (-0.12, 0.50) |
| Tertile 3 | 1.99 (1.68, 2.31) | 2.04 (1.73, 2.35) | 2.04 (1.73, 2.35) | 1.63 (1.26, 2.01) | 1.62 (1.25, 2.00) |
| P for trend | <0.001 | <0.001 | <0.001 | <0.001 | <0.001 |
| **Osteoporosis** |  |  |  |  |  |
| PAC (per 1-ng/dL increase) | 1.09 (1.06, 1.11) | 1.09 (1.06, 1.12) | 1.09 (1.06, 1.12) | 1.05 (1.02, 1.08) | 1.05 (1.02, 1.08) |
| Tertiles of PAC |  |  |  |  |  |
| Tertile 1 | Reference | Reference | Reference | Reference | Reference |
| Tertile 2 | 1.23 (0.85, 1.78) | 1.29 (0.88, 1.88) | 1.29 (0.89, 1.89) | 1.24 (0.83, 1.85) | 1.24 (0.83, 1.85) |
| Tertile 3 | 4.56 (3.27, 6.35) | 4.61 (3.27, 6.50) | 4.63 (3.28, 6.53) | 3.27 (2.14, 5.00) | 3.19 (2.08, 4.90) |
| P for trend | <0.001 | <0.001 | <0.001 | <0.001 | <0.001 |

Model 1: no covariates were adjusted.

Model 2: age, sex, BMI, smoking status, and drinking status were adjusted.

Model 3: Model 2 plus adjustment for DM, CHD, and cancer.

Model 4: Model 3 plus adjustment for ALT, AST, Cr, TC, TG, HDL-C, LDL-C, ALP, TSH, FPG, serum potassium, serum calcium, serum phosphorus, serum sodium, 24-h urinary potassium, 24-h urinary calcium,24-h urinary phosphorus, 24-h urinary sodium, PTH, and 25-hydroxyvitamin D.

Model 5: Model 4 plus adjustment for use of statins, aspirin, diuretics, beta-blockers, calcium channel blockers, ACEIs/ARBs, oral hypoglycemic agents, and insulin.

Abbreviations: PAC, plasma aldosterone concentration; BMD, bone mineral density; PA, primary aldosteronism; Neck, neck of the femur; Wards, Ward's triangle; MOF, major osteoporotic fracture; HF, hip fracture; β, regression coefficient; OR, odds ratio; CI, confidence interval.

Other abbreviations, see Table 1.

**References**

1. Farrell GC, Chitturi S, Lau GK, Sollano JD. Guidelines for the assessment and management of non-alcoholic fatty liver disease in the Asia-Pacific region: executive summary. Journal of gastroenterology and hepatology. 2007;22(6):775-7. Epub 2007/06/15. doi: 10.1111/j.1440-1746.2007.05002.x. PubMed PMID: 17565629.

2. Funder JW, Carey RM, Mantero F, Murad MH, Reincke M, Shibata H, et al. The Management of Primary Aldosteronism: Case Detection, Diagnosis, and Treatment: An Endocrine Society Clinical Practice Guideline. The Journal of clinical endocrinology and metabolism. 2016;101(5):1889-916. Epub 2016/03/05. doi: 10.1210/jc.2015-4061. PubMed PMID: 26934393.

3. Zhu Q, Heizhati M, Lin M, Wang M, Yao X, Gan L, et al. Higher Plasma Aldosterone Concentrations Are Associated With Elevated Risk of Aortic Dissection and Aneurysm: a Case-Control Study. Hypertension (Dallas, Tex : 1979). 2022;79(4):736-46. Epub 2022/01/13. doi: 10.1161/hypertensionaha.121.18342. PubMed PMID: 35016529.

4. Clemons M, Simmons C. Identifying menopause in breast cancer patients: considerations and implications. Breast cancer research and treatment. 2007;104(2):115-20. Epub 2006/10/25. doi: 10.1007/s10549-006-9401-y. PubMed PMID: 17061039.

5. Wen Z, Li Y, Xu L, Yue C, Wang Q, Chen R, et al. Triglyceride Glucose-Body Mass Index Is a Reliable Indicator of Bone Mineral Density and Risk of Osteoporotic Fracture in Middle-Aged and Elderly Nondiabetic Chinese Individuals. Journal of clinical medicine. 2022;11(19). Epub 2022/10/15. doi: 10.3390/jcm11195694. PubMed PMID: 36233562; PubMed Central PMCID: PMCPMC9572437.

6. Chan CY, Subramaniam S, Chin KY, Ima-Nirwana S, Muhammad N, Fairus A, et al. Levels of Knowledge, Beliefs, and Practices Regarding Osteoporosis and the Associations with Bone Mineral Density among Populations More Than 40 Years Old in Malaysia. International journal of environmental research and public health. 2019;16(21). Epub 2019/11/17. doi: 10.3390/ijerph16214115. PubMed PMID: 31731507; PubMed Central PMCID: PMCPMC6861980.

7. Yin RX, Wu DF, Miao L, Aung LH, Cao XL, Yan TT, et al. Several genetic polymorphisms interact with overweight/obesity to influence serum lipid levels. Cardiovascular diabetology. 2012;11:123. Epub 2012/10/09. doi: 10.1186/1475-2840-11-123. PubMed PMID: 23039238; PubMed Central PMCID: PMCPMC3508802.

8. Gao M, Lv J, Yu C, Guo Y, Bian Z, Yang R, et al. Metabolically healthy obesity, transition to unhealthy metabolic status, and vascular disease in Chinese adults: A cohort study. PLoS medicine. 2020;17(10):e1003351. Epub 2020/10/31. doi: 10.1371/journal.pmed.1003351. PubMed PMID: 33125374; PubMed Central PMCID: PMCPMC7598496.

9. Lv YB, Yuan JQ, Mao C, Gao X, Yin ZX, Kraus VB, et al. Association of Body Mass Index With Disability in Activities of Daily Living Among Chinese Adults 80 Years of Age or Older. JAMA network open. 2018;1(5):e181915. Epub 2019/01/16. doi: 10.1001/jamanetworkopen.2018.1915. PubMed PMID: 30646143; PubMed Central PMCID: PMCPMC6324469 Institutes of Health–National Institute of Neurological Disorders and Stroke outside the submitted work. No other disclosures were reported.
